# Expansion of forest cover and coeval shifts in Later Stone Age land-use at Taforalt and Rhafas Caves, Morocco, as inferred from carbon isotopes in ungulate tooth enamel

**DOI:** 10.1371/journal.pone.0325691

**Published:** 2025-06-12

**Authors:** Kayla B. Worthey, Philippe Fernandez, Elaine Turner, Teresa E. Steele, Louise Humphrey, R. Nick E. Barton, Jean-Jacques Hublin, Abdeljalil Bouzouggar

**Affiliations:** 1 American School of Prehistoric Research, Peabody Museum of Archaeology and Ethnology, Harvard University, Cambridge, Massachusetts, United States of America; 2 School of Anthropology, University of Arizona, Tucson, Arizona, United States of America; 3 CNRS, UMR 7269 LAMPEA, Aix Marseille Université, Ministry Culture, Aix-en-Provence, France; 4 Monrepos Archaeological Research Centre and Museum for Human Behavioural Evolution, LEIZA, Neuwied, Germany; 5 Department of Anthropology, University of California, Davis, California, United States of America; 6 Centre for Human Evolution Research, Natural History Museum, London, United Kingdom; 7 Institute of Archaeology, University of Oxford, Oxford, United Kingdom; 8 Paléoanthropologie, CIRB, Collège de France, Université PSL, CNRS, Paris, France; 9 Max Planck Institute for Evolutionary Anthropology, Leipzig, Germany; 10 Institut National des Sciences de l’Archéologie et du Patrimoine, Rabat, Morocco; Sapienza University of Rome: Universita degli Studi di Roma La Sapienza, ITALY

## Abstract

Later Stone Age (Iberomaurusian) hunter-gatherer groups in northwestern Africa appear to have experienced a major reorganization of land-use strategies and settlement dynamics around 15–13 cal ka BP, which broadly corresponds to the globally recognized Greenland Interstadial 1 (Bølling-Allerød) climate interval. However, our understanding of the local impacts of this interval on environments in Morocco is incomplete, as is our understanding of the strength of the relationship, if any, between paleoenvironmental change and human behavior in the Moroccan Later Stone Age. This paper reconstructs changes through time in local forest canopy cover during the Later Stone Age around the archaeological cave sites of Taforalt and Rhafas (northeastern Morocco), using stable isotopes of carbon in ungulate tooth enamel. Results indicate a close link between tree cover expansion during Greenland Interstadial 1 and changes in land-use behaviors, which at Taforalt included the exploitation of storable oak and pine-derived plant foods and greater intensity of site occupation. High local productivity of nut-bearing trees paired with regional increases in human population densities likely contributed to greater intensity of occupations at Taforalt and Rhafas during Greenland Interstadial 1.

## Introduction

The Later Stone Age (LSA) of northwestern Africa, associated with the Iberomaurusian technocomplex, spans 25–9 cal ka BP in Morocco, Algeria, Tunisia, and Libya, with the majority of sites dating to between 22 and 11 ka [1]. In addition to presenting a unique suite of stone tool technologies [2,3], organic tools [4–6], and symbolic artifacts [7,8], the LSA is characterized by more pronounced shifts in land-use than the preceding Middle Stone Age (MSA), including reorganizations of exploited resources and group mobility.

One regional shift in land-use patterns among LSA sites in northeastern Morocco is especially apparent between about 15–13 ka. Deposits in sites such as Taforalt, Ifri el Baroud, and Ifri n’Ammar dating to this interval show a marked increase in ash-dominated anthropogenic sedimentation and combustion. These features signal greater intensities of site occupation and fire use [9–12]. During this period cemeteries developed at sites such as Taforalt in Morocco and Afalou in Algeria, where at least 90 and 58 individuals were interred, respectively [13,14]. Taforalt also contains evidence of increased reliance on plants in human diets, demonstrated by the presence of ground stone [10], abundant macrobotanical remains [15,16] and evidence from stable isotopes of human teeth [17] and increased occurrences of dental caries [18]. Other evidence of diet broadening in northeastern Morocco comes from abundant land snail remains that often co-occur with ashy layers at Ifri el Baroud [12], Ifri n’Ammar [11,19], and Taforalt [20], in addition to contemporaneous mollusk consumption elsewhere in northern Africa such as at Abri Alain, Algeria [21] and Haua Fteah, Libya [22].

Furthermore, the interval between approximately 15–13 ka sees a spike in the number of LSA radiocarbon dates throughout northwestern Africa [1], indicating a widespread shift in settlement dynamics. After 13 ka, dated LSA archaeological occupations in the region are fewer. Taken together, the archaeological data suggest that between approximately 15–13 ka, LSA groups in Morocco were producing a more obvious and consequential human presence on the landscape through longer-duration site occupations, broadened diets, and greater investment into food processing.

These changes coincide with the end of the Heinrich Stadial 1 climate interval and beginning of Greenland Interstadial 1 (GI-1), or Bølling-Allerød, when around 14.7 ka numerous paleoclimate proxies indicate warming conditions in the western Mediterranean [23,24]. Increased moisture is suggested during this time as well, as pollen records indicate the expansion of arboreal habitats [25,26]. GI-1 is succeeded by the Younger Dryas, or Greenland Stadial 1 (GS-1), which is signaled by a return to cool, arid conditions in most western Mediterranean records starting around 12.5–13 ka [25,27].

Although limited, available paleoenvironmental evidence suggests variable vegetation response to climatic changes in Morocco during this period. Montane lake and wetland records from the Middle Atlas do not record major shifts in vegetation zones during GI-1, and instead see the greatest changes at the transition to Holocene climate regimes [28–30]. However, pollen data from the LSA site Ifri el Baroud in northeastern Morocco do suggest a local expansion of forested habitats during GI-1, coeval with intensified site occupations and resource use [12]. Meanwhile, at Taforalt, plant species represented in wood charcoal and phytoliths fluctuate throughout MIS 2 and may indicate a shift from juniper/thuja-dominated forest to one of pine and oak partway through GI-1 [15]. However, these proxies from archaeological contexts are filtered by human selection of firewood, vegetal foodstuffs, and plant fibers, which can obscure paleoenvironmental signals.

The temporal correspondence between changes in LSA land-use in northwest Africa between ca. 15–13 ka and the globally-recognized climate interval GI-1 raises questions about the relationship between the two phenomena. However, our understanding of 1) the actual local impacts of GI-1 on Moroccan landscapes, and 2) the synchronicity of human behavioral shifts with paleoenvironmental change, is incomplete. Only Ifri el Baroud [12] has produced environmental and archaeological proxies that change in concert over the GI-1 interval, and additional work is needed to determine if these trends are replicated at other sites.

To this end, we investigated local paleoenvironmental change at the sites of Taforalt and Rhafas in northeastern Morocco. Paleoenvironment was investigated through stable carbon isotope analyses of large herbivore tooth enamel. Stable isotopes in animal teeth directly reflect habitat changes in the foraging radii of the animals, which for the prey animals sampled here – Barbary sheep, gazelle, equines, and alcelaphines (primarily hartebeest) – are rarely estimated to be more than 15 km in regions outside of particular savannah settings such as the Serengeti [31–38]. While human foraging choices influence the areas from which animals are obtained and the relative abundances of prey species in the zooarchaeological assemblage, the isotope data obtained from animal teeth are otherwise independent environmental recorders of local to semi-local conditions. If climate change did prompt the suite of human behavioral shifts identified during the Later Stone Age, we expect to see evidence of local habitat remodeling concurrent with the modifications in forager land-use strategies seen in the archaeological records of Taforalt and Rhafas.

### Taforalt and Rhafas Caves

**Fig 1:**
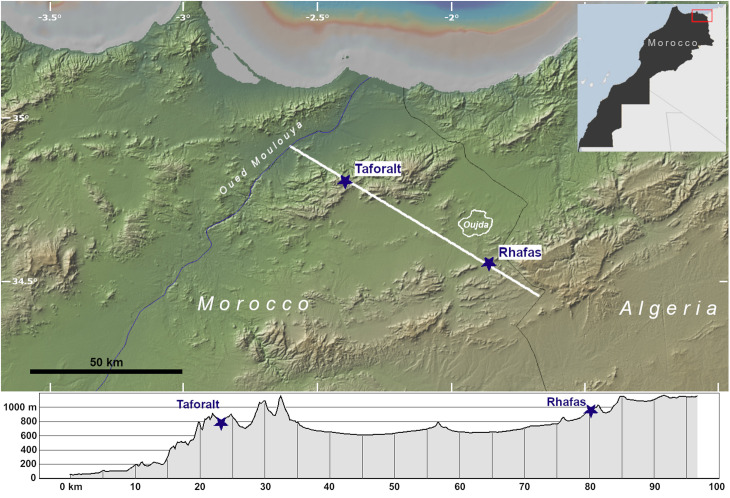
Locations of Taforalt and Rhafas caves in northeastern Morocco. The elevation profile is derived from a NW-SE transect indicated on the map by a white line, which runs from Oued Moulouya to the Algerian border and cross-cuts Taforalt and Rhafas Caves (blue stars). Figure made with GeoMapApp (www.geomapapp.org)/ CC BY/ CC BY [40].

The cave sites of Taforalt (Grotte des Pigeons) and Rhafas are located in northeastern Morocco, situated approximately 56 km apart on opposite sides of the Angads Plain, an inter-mountain basin where the city of Oujda is located (Fig 1). Taforalt is a northeast-facing cave within the Beni Snassen mountains (34° 48’ 50” N, 2° 24’ 14” W) close to one of a number of springs in the Zegzel Valley [10], while Rhafas [39] is a southeast-facing cave in the Oujda Mountains (34° 33’ 28’‘ N, 1° 52’ 26’‘ W). The sites are 40 and 50 km inland from today’s Mediterranean coast, respectively. Although they are at similar elevations (Taforalt: 720 m.a.s.l., Rhafas: 900 m.a.s.l.), the area around Taforalt is more topographically diverse, with mountain peaks as high as ~1200 m.a.s.l. and areas as low as ~20 m.a.s.l. in the Moulouya River Valley all located within a 20 km radius of the site. Meanwhile, Rhafas has access to comparable high elevation areas but the lowest areas accessible to the site in the Angads Plain are mostly 500 m.a.s.l. or more. Lastly, Rhafas is more proximal to flat topographic areas, while Taforalt is nestled deeper into the mountains, with the most easily accessible lowland area being the Moulouya River Valley.

Both sites have a long history of archaeological research. Major excavations at Taforalt began in 1944 with a series of investigations by A. Ruhlmann and then continued by J. Roche in several phases into the 1970s [41–45]. Additional work at the site was conducted in the 1980s by Raynal [46]. The most recent excavations at the site began in 2003 [9,10,47–49] and are ongoing, with 13 excavation sectors currently defined. Meanwhile, at Rhafas, preliminary investigations by J. Marion and J. Roche began in 1950 [42]. Formal excavations of the site by J. L. Wengler were undertaken in 1979 followed by several field seasons in the 1980s and 1990s [50–52]. Current work at the site began in 2007 and has continued intermittently in three areas: the lower cave, close to the cave mouth, and at the terrace outside of the cave drip line [39].

**Fig 2:**
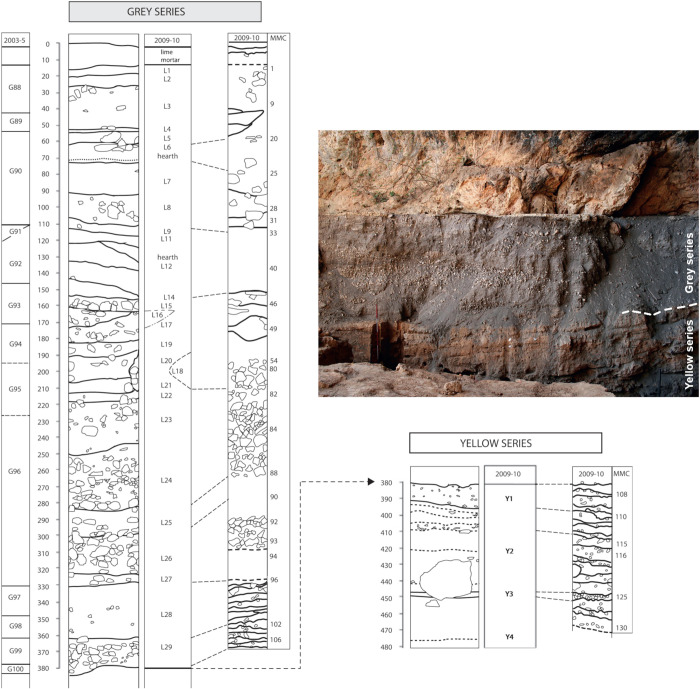
Taforalt Sector 8 stratigraphy of the Grey Series and Yellow Series. Figure modified from Humphrey et al. [18] S1 Fig and Collcutt [53] Fig 2.15. Photograph by Ian R. Cartwright.

Both Taforalt and Rhafas contain LSA occupations separated by an erosional unconformity into a chronologically earlier and later phase. At Taforalt, the earlier LSA component derives from a grouping of layers called the “Yellow Series” which is composed of finely laminated “clayey” sands and is present throughout much of the cave, being especially well-expressed in Sector 8 near the cave entrance [9]. The earliest secure LSA components in the Yellow Series date to around 22 ka. Around 15 ka, there is an irregular, erosional break followed by the deposition of extremely ashy, anthropogenic sediments termed the “Grey Series” (Fig 2). The sediments are predominantly massive or lenticular in nature, with high abundance of ash, charcoal, burned limestone, bone, and snail shell. Furthermore, the rate of sedimentation in the Grey Series was extremely rapid compared to the Yellow Series, with a deposition rate of approximately 1.7m/kyr [9]. The youngest Grey Series dates are around 12.6 ka, within the Younger Dryas climate interval (Table 1). All sediments above this were removed from the cave due to military activities in 1939 [10].

**Table 1:** Sampled contexts from Taforalt including the full range of ^14^C and OSL ages associated with each layer and assigned temporal intervals.

Sector	Layers	Date range (cal. years BP)	Dating method	Reference	Assigned temporal interval (this paper)	Notes
8	L3 (Grey series)	12,700−12,817	^14^C (n = 1). 2σ range of modeled posterior age	[54]	early Younger Dryas	
8	L6-L29/G100 (Grey series)	13,169−14,970	^14^C (n = 24). 2σ range of modeled posterior ages	[54]	GI-1 (Bølling-Allerød)	Sector 10 data exclude one sample (OxA-29264) with a modeled 2σ error range that does not overlap with the other samples
10	Grey series	13,993−15,211	^14^C (n = 16). 2σ range of ages	[54]		
10	Brown	Intermediate in age between Sector 10 grey series and Sector 13 orange layer	–	L. Humphrey personal communication (2024)	–	Although grouped here, differences in dental micro- and mesowear between these two layers may indicate they were deposited at slightly different times (Uzunidis et al., 2022)
13	Dark Brown					
8	Y1	14,855−15,615	^14^C (n = 4). 2σ range of modeled posterior ages	[5,4]	Heinrich Stadial 1	Excludes one sample (TAF08–6836) with a modeled 2σ error range that does not overlap with the other samples from layer Y1
13	Orange	17,569−17,982	^14^C (n = 1). 1σ range	[55]	Heinrich Stadial 1	^14^C date insecure given low collagen yield.
8	Y2	16,745−20,136	^14^C (n = 10). 2σ range of modeled posterior ages	[5,4]	Heinrich Stadial 1 to late LGM	
8	Y4	19,940−24,635	^14^C (n = 3). 2σ range of modeled posterior ages	[54]	LGM	
2	R7-R10	20,370−52,300	^14^C (n = 4) and OSL (n = 1). 1σ range of ages. Layers bracketed and not directly dated.	[5,5]	LGM or MIS 3	
8	Y8-Y13	30,880-34522	^14^C (n = 3). 2σ range of modeled posterior ages. Layers bracketed and not directly dated.	[9]	MIS 3	
2	R16-R17	56,200−64,000	OSL (n = 1). 1σ range	[5,5]	MIS 4	
2	R19-R20	56,200−88,900	OSL (n = 2). 1σ range. Layers bracketed and not directly dated.	[55]	MIS 4 or MIS 5a-b	
2	R21-R23	73,400−91,600	OSL (n = 1). 2σ range of modeled posterior age	[47]	MIS 5a-b	

The Taforalt Grey Series is also expressed at the back of the cave in Sector 10, where it contains at least 14 human burials [18,56,57]. The stratigraphical layer immediately underlying the Grey Series in Sector 10 is brown in color and not easily associated with the Yellow Series or Grey Series in other parts of the cave and thus will require additional dates to properly constrain its age. Similarly, in adjacent Sector 13 the stratigraphy consists of a dark brown layer and an underlying orange layer. The Sector 13 dark brown layer may be similar in age to the brown layer of Sector 10, or more likely is slightly older given that tooth micro- and mesowear indicate differences in the dietary signals of ungulates in the two layers [58]. The orange layer produced an unpublished radiocarbon date on bone of ~17.8 cal. ka BP that is potentially unreliable due to low collagen yield but suggests contemporaneity with the Yellow Series. Sector 2 of the site preserves a longer temporal sequence into the MSA, with OSL and U-series dating providing an age of ~112.7 ka in the deepest layers indicating that the earliest occupation of the site took place during MIS 5d [59].

**Fig 3:**
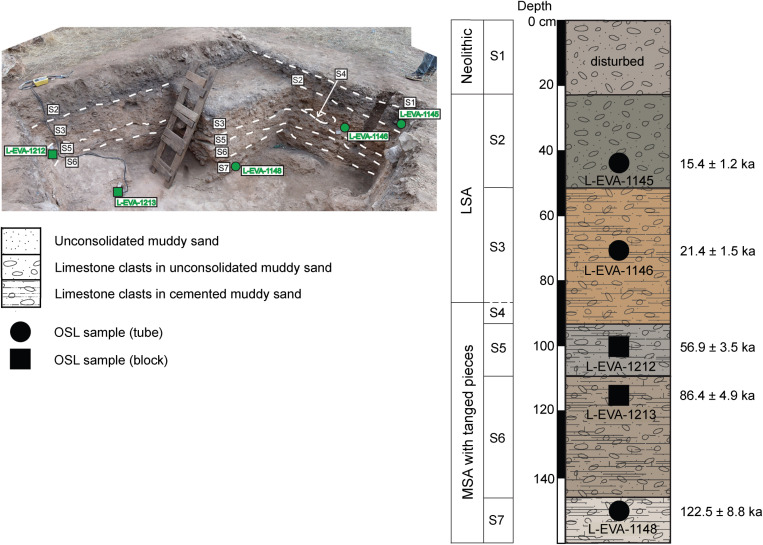
Rhafas terrace section stratigraphy. including locations of OSL samples. Figure modified from Doerschner et al. [39] Fig 2e and 8b.

In Rhafas, the excavated LSA layers are restricted to the terrace section of the site in layers S2 and S3 [39]. The earlier LSA layer S3 is characterized by cemented reddish grey sediments with frequent clasts of limestone. A single OSL date of 21.4 + /- 1.5 ka suggests that the layer was deposited at least in part during the Last Glacial Maximum. An erosional unconformity truncates layer S3 and is overlain by layer S2, which contains less cemented, dark grey sediments with an important anthropogenic contribution of ash (Fig 3). An OSL date of 15.4 + /- 1.2 ka, while slightly early, places layer S2 within error of GI-1 and the Grey Series documented at Taforalt (Table 2). While additional study is required to better develop the chronology for the LSA deposits at Rhafas, the presence of an erosional unconformity followed by ash-dominated deposits is consistent with that seen at Taforalt and other sites in northeastern Morocco including Ifri n’Ammar and Ifri el Baroud. We hypothesize that this pattern is part of a regional and broadly contemporaneous phenomenon in the later LSA expressed as increased human presence on the landscape with frequent and extensive fire use within cave sites.

**Table 2:** Sampled contexts from Rhafas including associated OSL ages following Doerschner et al. [39] and assigned temporal intervals.

Sector	Layer(s)	OSL age (1σ)	Date range (cal. years BP)	Assigned temporal interval (this paper)
Terrace	S2	15.4 ± 1.2 ka	14,200 − 16,600	GI-1 (Bølling-Allerød)
Terrace	S3	21.4 ± 1.5 ka	19,900 − 22,900	LGM
Terrace	S5	56.9 ± 3.5 ka	56,900−60,400	early MIS 3 or late MIS 4
Terrace	S7	122.5 ± 8.8 ka	113,700−131,300	MIS 5d-5e
Cave mouth	4c	135.3 ± 10.3 ka	125,000-145,600	MIS 5e or MIS 6
Lower cave	5 - 55	>135.3 ± 10.3 ka	>125,000-145,600	MIS 5e, MIS 6, or older

Deeper excavated layers in the terrace and cave mouth at Rhafas contain MSA occupations and date by single-grained OSL to as early as MIS 5e [39]. OSL ages do not continue to increase with depth in the lower cave section, hypothesized to be caused by post-depositional infiltration of radioactive elements in this part of the cave. The lower cave section contains approximately 2 meters of excavated sediments divided into 55 layers by Wengler [50], and although dating by OSL has been unsuccessful, they are presumed to date to at least MIS 5e and are likely older [39].

Despite being located in the same region of Morocco east of the Rif, the particularities of the Taforalt and Rhafas site environs supported different communities of large herbivores. At Taforalt, Barbary sheep (*Ammotragus lervia*) is by far the best represented taxon in the zooarchaeological assemblage, while at Rhafas, equines (zebra/*Equus* cf. *mauritanicus,* wild ass/*Equus* cf. *africanus*) dominate. This difference in archaeofaunal composition is consistent with modern habitat preferences for these or closely related taxa; Barbary sheep today prefer areas with steep slopes and large altitudinal gradients as is seen near Taforalt, and frequently occupy habitats that include a forest component [60,61]. Zebras and wild ass prefer grassy plains [38], which would have been more accessible to Rhafas foragers. Both sites also contain gazelles (*Gazella* sp.) in lower numbers, which in northwestern Africa tend to occupy mountains and hilly zones [62,63]. Alcelaphines, including hartebeest/*Alcelaphus buselaphus*, present in both sites, may occupy forested areas when not feeding but prefer to forage in open grassy habitats and are more similar to equines in this regard [62]. Micro- and mesowear studies of ungulates from Taforalt confirm these habitat preferences, with Barbary sheep and gazelles presenting dental wear consistent with a browse-dominated diet, while wear patterns in alcelaphines and equines indicate mixed and grazing-dominated diets respectively [58].

### Carbon isotopes in ungulate teeth

Stable carbon isotopes (henceforth δ^13^C values) in the carbonate component of tooth enamel reflect the average δ^13^C values of carbon in the animal’s diet. In herbivore ungulates, carbon is derived principally from plant leaves, stems, inflorescences, and fruits. First-order variation of vegetation δ^13^C values on a landscape is driven by the photosynthetic pathway employed by the plants. Globally, modern plants utilizing C4 photosynthesis have average δ^13^C values between −14 to −10‰ plants using C3 photosynthesis have a typical δ^13^C value range between −31.5 and −23‰, and plants using crassulacean acid metabolism (CAM) have intermediate δ^13^C values [64,65].

C4 photosynthesis evolved independently in multiple plant lineages but is most common in warm-season grasses. As such, C4 plant abundance on the landscape is primarily controlled by the seasonal distribution of rainfall [66–68]. Summer precipitation will facilitate the growth of C4 grasses, while winter-dominated precipitation, as is seen in Mediterranean climates, favors C3 plants. C4 grass can also out-compete C3 plants under conditions of lower atmospheric CO_2_ concentrations (pCO_2_). If present on the landscape, C4 grasses are more likely to be consumed by grazers and mixed feeders rather than herbivores that favor browse [69].

Second-order controls on δ^13^C values in ungulate teeth include the δ^13^C values of atmospheric CO_2_ (δ^13^C_atm_) [70], pCO_2_ [71], and the level of moisture available to C3 plants [65]. Variation in moisture availability accounts for much of the wide δ^13^C value range observed globally in modern C3 plants, with plants experiencing water deficit expressing higher plant δ^13^C values. Water stress is reduced if plants receive more precipitation [65], are situated in slower draining soils [72], are growing under cooler temperatures [73], or are experiencing lower intensities of light. Among C3 plants within the same habitat, light intensity as controlled by canopy cover seems to be one of the driving sources of δ^13^C value variability [74,75]. This is not to be confused with the “canopy effect” which is another mechanism that substantially decreases δ^13^C values in plants that are utilizing recycled CO_2_ released from decaying plant litter where extremely dense forest canopies prevent its dissipation. Lower δ^13^C values due to reduced light intensity is a widespread phenomenon in temperate forests and mixed woodland/grassland habitats, whereas the “canopy effect” *sensu stricto* has only been documented in dense tropical rainforests [76,77].

At Taforalt and Rhafas, carbon isotopes will be examined in grazers (equines, alcelaphines) and mixed feeders (Barbary sheep, gazelles) to measure change through time in the environmental aspects most readily detected by the proxy: namely, C4 plant incursions and variation in water stress among C3 plants. This study aims to test whether plant communities and/or degrees of plant water stress change in the archaeological layers that span known shifts in the land-use strategies of foragers in northwestern Africa. Additionally, since the species sampled have somewhat different forage and habitat preferences, carbon isotopes have the potential to reveal habitat-specific spatial trends in vegetation change.

## Materials and methods

A combined sample of 111 herbivore teeth from Taforalt (n = 56) and Rhafas (n = 55) were selected for isotope analysis. The dental samples represent four ungulate genera: Barbary sheep (*Ammotragus lervia*), equines (*Equus* sp. including *Equus africanus* and *Equus mauritanicus*), gazelles (*Gazella* sp.), and alcelaphines (Alcelaphini including *Alcelaphus buselaphus*/hartebeest). The sample from Taforalt is dominated by Barbary sheep (n = 39; 70%) (Table 3) while the sample from Rhafas is equine-dominant (n = 33; 60%) (Table 4), reflecting the differential species representation in the archaeofaunas from the two sites.

**Table 3:** Sampled specimens from Taforalt by layer and taxon.

Sector	Layer	Barbary sheep (*Ammotragus lervia*)	Equine (*Equus* sp.)	Alcelaphine (Alcelaphini)	Gazelle (*Gazella* sp.)	Total
Sector 2	R7-R10	3				**3**
	R16-R17	6	1			**7**
	R19-R20			2		**2**
	R21-R23	3	3		1	**7**
Sector 8	L3 (Grey series)	1				**1**
	L6-L29 (Grey series)	12	1		1	**14**
	Y1	1				**1**
	Y2	4				**4**
	Y4	3				**3**
	Y8-Y13	2				**2**
Sector 10	Grey		1			**1**
	Brown		2			**2**
Sector 13	Dark brown	1			1	**2**
	Orange	3	2	1	1	**7**
	**Total**	**39**	**10**	**3**	**4**	**56**

**Table 4:** Sampled specimens from Rhafas by layer and taxon.

Sector	Layer	Barbary sheep (*Ammotragus lervia*)	Equine (*Equus* sp.)	Alcelaphine (Alcelaphini)	Gazelle (*Gazella* sp.)	Total
Terrace	S2	2	6	9	4	**21**
	S3		3			**3**
	S5		1			**1**
	S7		6			**6**
Cave Mouth	4c		3		1	**4**
Lower Cave	5-55		14	5	1	**20**
	**Total**	**2**	**33**	**14**	**6**	**55**

Due to limited availability of specimens, the sample includes mandibular and maxillary incisors, 2^nd^, 3^rd^, and 4^th^ premolars, 1^st^, 2^nd^, and 3^rd^ molars, and unassigned premolars and molars (S1 Table). While nursing and weaning can affect the δ^13^C values of tissues among human infants, polar bear, and pinniped young on the order of 1‰ or less [78–80], this is not observed among terrestrial herbivores [81,82]. Therefore, unlike δ^15^N (and occasionally δ^18^O) values [82], δ^13^C values in tooth enamel apatite that formed early in life (such as 1^st^ molar enamel) would not be measurably affected by nursing and weaning, particularly since weaning diets are expected to be comparable with the maternal diet [83,84]. However, differences in the timing and duration of tooth formation in this heterogeneous sample may introduce seasonal variability into the isotope results. This variability could obscure climate-mediated patterns in the isotope data. To quantify the scale of potential seasonal variability in δ^13^C values, a subset (n = 30) of teeth were sequentially sampled following previously published protocols [85,86]. The annual range of δ^13^C values averages 1.0 ± 0.3‰ in the sample (S2 Table, S1 File), and any “noise” due to uneven sampling of seasons is not expected to exceed this value. Thus, any detected variation in carbon isotope values on the order of 1‰ or more are expected to be reliable indicators of environmental conditions or types of vegetations that were consumed.

Teeth were cleaned and the outer layer of enamel removed by abrasion with a tungsten-carbide drill bit mounted in a rotary tool. A 0.6–0.8 mm furrow was drilled from the cervix to the apex of the crown parallel to the growth axis of the tooth and through the entire enamel thickness. Clean enamel powder was collected on weighing paper. To test the effect of acetic acid pretreatment on the samples, a subset of representative samples from Taforalt (n = 12) and Rhafas (n = 14) were submerged for 30 minutes in 0.1 M acetic acid. Samples were rinsed repeatedly with deionized (DI) water until neutrality was reached and dried overnight at 50°C. Differences between untreated and pretreated sample δ^13^C values were minimal, averaging −0.20‰ and −0.21‰ at Taforalt and Rhafas respectively, with similar offsets observed in all layers at the sites (S3 Table). In this paper, δ^13^C values from untreated enamel are used due to the minimal and consistent observed offsets between untreated and pretreated samples, combined with concerns about the structural integrity of pretreated enamel powders raised by Spencer et al. [87] and Varkuleviciute et al. [88], among others. For sequentially sampled teeth, mean δ^13^C values of the subsamples are used for analysis.

Stable isotope analyses were carried out at the University of Arizona Environmental Isotope Laboratory in Tucson, AZ. δ^13^ C (δ^13^ C_enamel_ ) and δ^18^ O (δ^18^ O_enamel_ ) values were measured using an automated carbonate preparation device (KIEL-III) coupled to a gas-ratio mass spectrometer (Finnigan MAT 252). A subsample of 1–2 mg powdered enamel from each specimen was reacted with dehydrated phosphoric acid under vacuum at 70°C in the presence of silver foil. The isotope ratio measurement is calibrated based on repeated measurements of NBS-19 and NBS-18, and precision is ± 0.10‰ for δ^18^O and ±0.08‰ for δ^13^C (1 sigma). Carbon and oxygen isotope results of enamel carbonate are reported in delta notation (δ‰) relative to the VPDB standard. The carbonate–CO_2_ fractionation for the acid extraction is assumed to be identical to calcite.

The δ^13^C_enamel_ values of ungulates with different digestive physiologies are not directly comparable, because digestion processes affect enamel-diet fractionation factors. To reconstruct dietary δ^13^C (δ^13^C_diet_ ) values, we follow Cerling et al. [89] and assign isotope enrichment values (ε_enamel-diet_ values) of 14.5 ± 1‰ to ruminant herbivores (Barbary sheep, gazelles, alcelaphines) and 13.5 ± 1‰ to non-ruminant herbivores (equines). Statistical t-tests (two-tailed) were performed using the Data Analysis package in Excel, and in all cases assume unequal sample variances.

The archaeofaunal remains sampled in this study are curated at the Institut National des Sciences de l’Archéologie et du Patrimoine (INSAP) in Rabat, Morocco. Permits to sample and analyze enamel powders for stable isotopes were issued by INSAP. All necessary permits were obtained for the described study, which complied with all relevant regulations.

## Results

**Fig 4:**
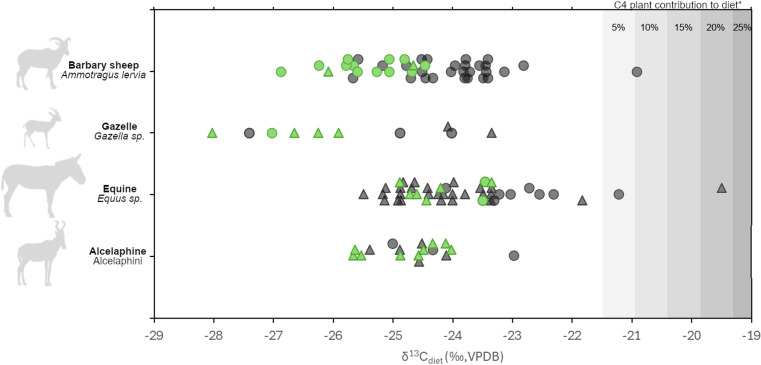
Reconstructed δ^13^C values of vegetation in the diet (δ^13^C_diet_) of Barbary sheep, gazelle, equines, and alcelaphines from Taforalt (circles) and Rhafas (triangles), following δ^13^C_enamel-diet_ fractionation factors published in Cerling et al. [89]. Green symbols indicate specimens derived from LSA contexts thought to be coeval with the GI-1/Bølling-Allerød interstadial. GI-1 is associated with lower δ^13^C_diet_ values in Barbary sheep and gazelle, but not in equines or alcelaphines. *C4 plant contributions to diet, indicated in grey shading, should be considered conservative estimates given that the chosen C3 plant endmember (−21.5 ‰ VPDB) is for a C3 plant in an arid climate [65]. An atmospheric correction of +1.5 ‰ was applied to C3 and C4 plant endmembers.

Measured δ^13^C_enamel_ values and reconstructed δ^13^C_diet_ values average −10.3 ± 1.1‰ and −24.5 ± 1.1‰ (VPDB) respectively for Barbary sheep, −11.6 ± 1.6‰ and −25.8 ± 1.6‰ for gazelles, −10.7 ± 1.2‰ and −23.9 ± 1.2‰ for equines, and −10.5 ± 0.7‰ and −24.7 ± 0.7‰ for alcelaphines. These values indicate the predominance of C3 plants on the landscape in the vicinity of Taforalt and Rhafas. Of 111 sampled specimens, only three (1 equine from Rhafas, 1 equine from Taforalt, 1 Barbary sheep from Taforalt) have a δ^13^C_diet_ value above −21.5‰ indicating a C4 plant contribution to their diet. In these cases, C4 plants make up a relatively low proportion of the diet: ~ 10% or less in two of three individuals, and less than 25% in the third (Fig 4). Among sequentially sampled specimens, seasonal variation in δ^13^C_enamel_ values averages 1.1 ± 0.35‰ (VPDB) and is relatively consistent between taxa (S2 Table).

**Fig 5:**
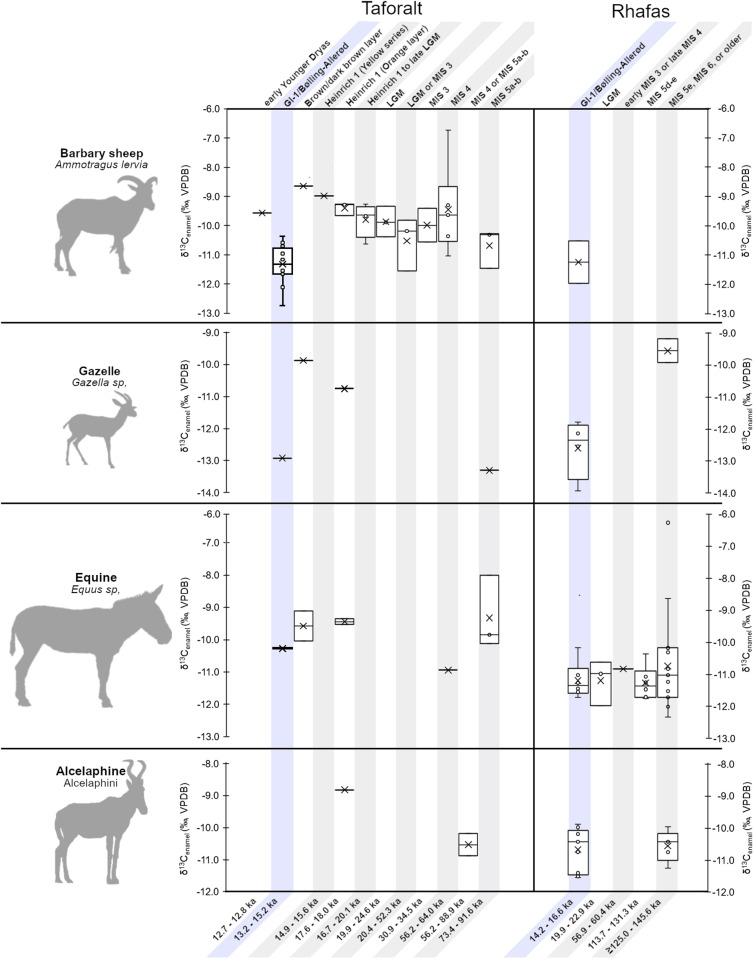
δ^13^C_enamel_ values among Barbary sheep, gazelle, equines, and alcelaphines at Taforalt (left) and Rhafas (right), separated by temporal-stratigraphic interval with dates followingTables 1 and 2. Barbary sheep and gazelle δ^13^C_enamel_ values show negative excursions during the GI-1 (Bølling-Allerød) interval (highlighted in blue shading).

The four species present a similar range of δ^13^C_diet_ values, with the notable exception of Barbary sheep and gazelle specimens with δ^13^C_diet_ values that derive from layers dating to or within error of Greenland Interstadial 1 (GI-1) (Table 5, Figs 4 and 5). Barbary sheep from these layers have statistically lower δ^13^C_diet_ values than those from other LSA and earlier MSA contexts (t(34)=−5.77, p < 0.001) differing by 1.5‰ (VPDB). The same is true of gazelles (t(6)=−2.54, p < 0.05), which have δ^13^C_diet_ values that are 2.1‰ (VPDB) lower in the GI-1 interval. In contrast, no statistical differences are observed δ^13^C_diet_ values during GI-1 for equines (t(22)=−1.16, p = 0.26), nor for alcelaphines (t(14)=−0.98, p = 0.34).

**Table 5:** Summary statistics of δ^13^C_enamel_ and δ^13^C_diet_ values of specimens dating to GI-1 in comparison to those from other periods. Data are arranged by taxon and site. The final two rows for each taxon combine specimens from Taforalt and Rhafas. An additional row with data from the Younger Dryas (YD) and Heinrich Stadial (HS) 1 is included for Barbary sheep specimens from Taforalt, to illustrate Barbary sheep δ^13^C_enamel_ and δ^13^C_diet_ values in the contexts immediately pre- and post-dating GI-1. T-test p-value results are further discussed in the text.

				δ^13^C_enamel_(‰, VPDB)				δ^13^C_diet_ (‰, VPDB)				
Taxon	Site	Assigned temporal interval	N	Mean	stdev	Median	Range	Mean	stdev	Median	Range	p-value*
Barbary sheep	Taforalt	GI-1 (Bølling-Allerød)	12	−11.31	0.70	−11.30	2.45	−25.44	0.69	−25.44	2.42	<0.001
		Younger Dryas & Heinrich Stadial 1	5	−9.35	0.27	−9.29	0.67	−23.51	0.26	−23.45	0.66	
		YD, HS 1, and other periods	27	−9.79	0.94	−9.65	4.82	−23.95	0.93	−23.80	5.16	
	Rhafas	GI-1 (Bølling-Allerød)	2	−11.24	1.02	−11.24	1.45	−25.37	1.01	−25.37	1.43	
		Other periods	–	–	–	–	–	–	–	–	–	
	All GI-1 (Bølling-Allerød)		14	−11.30	0.70	−11.30	2.45	−25.43	0.69	−25.44	2.42	<0.001
		All other periods		27	−9.79	0.94	−9.65	5.23	−23.95	0.93	−23.80	4.76
Gazelle	Taforalt	GI-1 (Bølling-Allerød)	1	−12.92	–	−12.92	–	−27.03	–	−27.03	–	
		Other periods	3	−11.31	1.78	−10.75	3.43	−25.44	1.76	−24.89	3.38	
	Rhafas	GI-1 (Bølling-Allerød)	4	−12.60	0.94	−12.34	2.14	−26.71	0.93	−26.46	2.11	
		Other periods	2	−9.56	0.53	−9.56	0.74	−23.71	0.52	−23.71	0.73	
	All GI-1 (Bølling-Allerød)		5	−12.66	0.83	−12.54	2.14	−26.78	0.81	−26.66	2.11	<0.05
		All other periods		5	−10.61	1.61	−9.93	4.12	−24.75	1.58	−24.08	4.06
Equine	Taforalt	GI-1 (Bølling-Allerød)	2	−10.30	0.02	−10.30	0.04	−23.48	0.02	−23.48	0.03	
		Other periods	8	−9.62	0.86	−9.69	2.93	−22.81	0.85	−22.88	2.89	
	Rhafas	GI-1 (Bølling-Allerød)	6	−11.20	0.56	−11.36	1.55	−24.37	0.55	−24.52	1.53	
		Other periods	27	−10.91	1.22	−11.07	6.07	−24.08	1.21	−24.25	5.99	
	All GI-1 (Bølling-Allerød)		8	−10.97	0.63	−11.15	1.55	−24.15	0.62	−24.32	1.53	0.26
		All other periods		35	−10.61	1.26	−10.83	6.07	−23.79	1.25	−24.01	5.99
Alcelaphine	Taforalt	GI-1 (Bølling-Allerød)	–	–	–	–	–	–	–	–	–	
		Other periods	2	−9.96	1.05	−10.19	2.06	−24.11	1.03	−24.34	2.03	
	Rhafas	GI-1 (Bølling-Allerød)	9	−10.67	0.66	−10.43	1.66	−24.81	0.65	−24.57	1.64	
		Other periods	5	−10.55	0.48	−10.42	1.30	−24.69	0.48	−24.57	1.28	
	All GI-1 (Bølling-Allerød)		9	−10.67	0.66	−10.43	1.66	−24.81	0.65	−24.57	1.64	0.34
		All other periods		7	−10.33	0.74	−10.40	2.44	−24.48	0.72	−24.54	2.40

*p-values derive from two-tailed t-tests assuming unequal variances performed on δ^13^C_enamel_ values.

A detailed look at δ^13^C_enamel_ values through time in Taforalt and Rhafas (Fig 5) reveals that, although several layers contain only single specimens, Barbary sheep δ^13^C_enamel_ values from GI-1 (n = 14) are statistically lower than those immediately pre- or post-dating GI-1, which include specimens from Heinrich Stadial 1 (n = 4) and the sample from the following Younger Dryas climate interval (t(17)=−8.76,p < 0.001). The magnitude of this shift is 2.0‰ (VPDB). The specimen from the dark brown layer in Taforalt is poorly dated but is isotopically similar to specimens in layers that pre-date GI-1. Meanwhile, although the MIS 5a-b interval represented at Taforalt contains a small sample of Barbary sheep and gazelle teeth (n = 3 and n = 1, respectively), δ^13^C_diet_ values for these taxa tend to be low in these layers and similar in value to those from GI-1. Other taxa are not sufficiently abundant to support fine-grained temporal comparisons with the exception of the Rhafas equines, which display relatively stable δ^13^C_enamel_ values through time.

Meanwhile, δ^13^O_enamel_ values are highly variable, averaging 0.37 ± 1.7‰ (VPDB) for Barbary sheep, 0.31 ± 1.6‰ for gazelle, −2.9 ± 1.0‰ for equines, and −1.87 ± 1.3‰ for alcelaphines. Inter-species differences in δ^13^O_enamel_ values are likely driven by different proportions of water ingested via plant water and drinking from meteoric water sources. Visual examination of temporal patterning of δ^13^O_enamel_ values reveal similar directionalities among all taxa, with lowest values among the oldest (≥MIS 5e) contexts at Rhafas, and generally higher values in LGM contexts at the two sites (S1 Table, S1 Fig).

## Discussion

Among the four taxa studied, the low δ^13^C value excursion in the GI-1 interval at Taforalt and Rhafas is expressed only by Barbary sheep and gazelles. The former (*A. lervia*) naturally prefers montane and hilly environments [60,61] while *Gazella* frequents a range of habitats from deserts to mountainous settings [62]. In Taforalt, *G. cuvieri* is likely to be represented among the sampled gazelle specimens given its confirmed presence at the site [90]. Like *A. lervia,* this species is frequently associated with a rather woody plant cover [91]. Both taxa were likely inhabiting the environment close to Taforalt, consuming a browse-dominated or mixed diet. Meanwhile, equines and alcelaphines (primarily hartebeest) were more likely encountered in the lowland grassy plains accessible to both sites, and particularly close to Rhafas. Their δ^13^C_diet_ values indicate that in the layers sampled their habitats were made up of C3, rather than C4, grasslands, as would be typical of regions with winter-dominated rainfall regimes. The availability of C3 grasses has also been confirmed at Taforalt from the presence of *Stipa tenacissima* rhizome fragments in LSA layers [16].

Since the δ^13^C value excursion in GI-1 is not universally observed among the four taxa, fluctuations in global pCO_2_ and δ^13^C_atm_ cannot reasonably account for the shift. A dietary change among Barbary sheep and gazelles during GI-1, while possible, is unlikely to be the only cause of their lower δ^13^C_diet_ values during this interval. C4 plants are rare in the study area and in the diets of sampled individuals, so changing abundances of C3 and C4 plants do not explain the pattern. Leafy browse has sometimes been cited as having δ^13^C values 1−2‰ lower than C3 grasses [70] (but see [92,93]). However, tooth microwear and mesowear information from Taforalt [58] in combination with the stable isotope data suggest that plant functional type was not the most important factor structuring δ^13^C value variability in the area. The tooth wear results indicate that, in layers associated with Heinrich 1, Barbary sheep and gazelles were consuming browse, while equines and hartebeest were consuming grasses [58]. Yet, average browser and grazer δ^13^C_diet_ values during H1 differ by only 0.7‰ (VPDB). This is not enough to account for the GI-1 shift in δ^13^C values of Barbary sheep and gazelles, which is a magnitude of 2.0‰ (VPDB) lower among Barbary sheep relative to immediately preceding and following periods, and an average of 1.5‰ and 2.1‰ lower among Barbary sheep and gazelles, respectively, relative to all other sampled periods (Table 5).

Instead, the anomaly is better interpreted as a “wet” signal among C3 plants under conditions that affected mountains and hills where Barbary sheep and gazelles foraged but *not* the grassy plains that supported equines and alcelaphines. Several potential explanations related to plant δ^13^C value variability as a function of water stress are considered in sequence. First, given that Barbary sheep and gazelles consume greater amounts of browse plants relative to equines and alcelaphines, it is reasonable to ask if browse may be more isotopically responsive to changes in mean annual precipitation than grasses. However, a large body of published literature demonstrates that δ^13^C values in C3 grass do respond to moisture differences at similar magnitudes [94–96].

Second, the relationship between δ^13^C values and water stress in plants is stronger for plants in arid or highly evaporative environments, and weaker for plants that are already well-watered [65]. It is therefore important to consider if grasses near Taforalt and Rhafas were growing under moister conditions that buffered them from changes in mean annual precipitation. If that was the case, we would expect lower δ^13^C_diet_ values among grass-eating equines and alcelaphines relative to Barbary sheep and gazelles from periods preceding or post-dating GI-1; however, we do not find this pattern (Fig 4).

Third, it is important to consider that shifts in Barbary sheep and gazelle altitudinal ranges and/or changes in the hunting areas targeted by LSA foragers may account for the lower δ13C_diet_ values that are observed at Taforalt during GI-1. Seasonal range expansions among modern Barbary sheep tend to be lateral rather than altitudinal [32,35], but this does not necessarily exclude the possibility of altitudinal shifts in the past at these sites. To evaluate this possibility, it is useful to consider how much δ^13^C values would be expected to change over the local altitudinal gradient. At elevations of 2,000 meters above sea level (masl) or less in a water-limited environment such as the Mediterranean, it is reasonable to hypothesize that higher elevations would associate with lower δ^13^C values due to gradients in rainfall. Meanwhile, above ~2,000 masl, δ^13^C values decrease with altitude due to declining pCO_2_ [97]. In the Ethiopian Rift Valley, which has particularly strong altitudinal zonation of precipitation due to its vertical extent (900 to >4,000 masl), an approximate 2‰ (VPDB) decrease occurs in the δ^13^C values of plant leaves between 1,000 and 2,000 masl [98]. Using this point of reference, the 2‰ shift in δ^13^C values observed in Barbary sheep from Taforalt during GI-1 (Figs 4 and 5) would require animal home ranges and/or hunter procurement areas to shift from the base of the Beni Snassen Mountains to their summit. This scenario is unlikely, particularly because faunal analysis indicates that body part representation of Barbary sheep at Taforalt show no changes through time. Body part representation is consistent with the transport of whole carcasses to the site both during and prior to GI-1 [90], indicating that Barbary sheep were obtained sufficiently close to the site that no field processing occurred to reduce transport weight.

Instead, the most parsimonious scenario to explain the observed GI-1 decrease in δ^13^C values among Barbary sheep and gazelles is forest expansion and canopy in-filling in the higher elevation environments that these species exploit. Light intensities due to variable canopy cover in temperate environments have been shown to have large effects on plant δ^13^C values, on the order of 4−6‰ [75]. During GI-1, reduced light intensities in the understories would have lowered δ^13^C values among plants accessible to browsing and mixed-feeding herbivores. Meanwhile, equines and alcelaphines would have fed in open habitats where light intensities remained consistently high. The trees around the sites today are dominated by thuja/*Tetraclinis articulata*, Aleppo pine/*Pinus halepensis,* and Holm oak/*Quercus ilex* [47,99]. At present the tree leafy biomass is sufficiently expansive to form shaded canopies in many areas around Taforalt, but these species are also known to respond to water or temperature stress by reducing their growth rate, canopy extent, and productivity. Due to their adaptive plasticity, it is possible for these tree species to persist through different climate regimes but with varying degrees of canopy cover [10]. This interpretation would be consistent with independent pollen evidence from Ifri El Baroud, which indicates arboreal expansion in northeastern Morocco during GI-1 [12].

Forest canopy in-filling likely would relate to reduced drought and freeze stress experienced by trees [100], which is consistent with increased precipitation amounts and warm temperatures during GI-1. Although these climate factors would have been felt regionally, the current study indicates that light intensity as a function of canopy cover may have influenced vegetation δ^13^C values more than precipitation amount or temperature. This may be related to the fact that precipitation amount has a negative relationship with vegetation δ^13^C values while temperature (by raising potential evapotranspiration) has a positive one, so that increases in both would tend to offset their influence on vegetation δ^13^C values. Finally, it remains possible that reduced light intensities at higher elevations worked to lower δ^13^C values in Barbary sheep and gazelles in concert with other, previously discussed factors that alone do not account for the shift, such as dietary changes or home range shifts.

Unlike δ^13^C, δ^18^O_enamel_ values show similar temporal trends among all sampled species, suggesting that they are primarily patterned by a different set of variables such as the δ^18^O values of precipitation, even when taxa such as Barbary sheep and gazelles are positively offset from equine and alcelaphine δ^18^O_enamel_ values due to their proportionally greater intake of leaf water (S1 Table, S1 Fig). Additional Barbary sheep, gazelle, and equine samples from LGM and MIS 3 contexts would be required to assess whether evaporative enrichment of leaf waters was greater during this interval, as is seen in the late MIS 3 contexts of Gebel Akhdar, Libya [101].

This study serves to not only identify canopy cover as an important environmental factor during GI-1, but it also helps to relate the timing of environmental changes to specific archaeological layers and cultural developments. Previous paleoenvironmental data from Taforalt suggested potentially drier conditions at the very base of the Grey Series relative to what followed thereafter. Evidence for this are remains of Barbary ground squirrel (*Atlantoxerus getulus*) and Moorish gecko (*Tarentola mauritanica*), together with a greater juniper/thuja charcoal component at the base of the Grey Series, with more pine and oak charcoal later in time [10]. However, this study suggests the increase in canopy cover corresponds sharply with the beginning of the Grey Series, with tooth specimens coming from the lower-most Grey Series layers (28–29/G98-G100) already displaying anomalously low δ^13^C values. Furthermore, our data suggest that the brown layers in Sectors 10 and 13 of Taforalt, which are not directly dated, separate isotopically from the Grey Series and so may in fact pre-date it.

Independent lines of evidence derived from Taforalt’s rich archaeological record are consistent with an increase in forest resource productivity during GI-1. Acorn and pine nut food refuse make up much of the rich macrobotanical record in the Grey Series [10,16]. Intense acorn consumption, in particular, is indicated by the presence of ground stone suitable for acorn processing, a high prevalence of dental caries in the Taforalt human remains [18], and stable isotope evidence of plant-dominated diets among site occupants [17]. Acorns are starchy foods that produce fermentable carbohydrates that can lead to advanced enamel demineralization and periodontal disease, particularly when ground or cooked before consumption. Increased use of fire evidenced in the Grey Series from ash, charred materials, and fire-cracked rocks [10] may also relate to extensive food processing activities, such as the charring and opening of pinecones or the drying of plant foods and meat for storage.

Acorns and pine nuts lend themselves well to storage for later consumption. Natural concentrations of oak and pine trees around Taforalt, and potentially other contemporary sites, made these caves especially attractive to LSA foragers. Macrobotanical and phytolith evidence for esparto grass (*Stipa tenacissima*) at Taforalt is consistent with the use of woven technologies for storage, given the grass’s uses in basketry today [16,18], as is the presence of specialized bone tools for weaving [5,6]. Storage practices tend to associate with longer stays at sites, by effectively increasing the resource yield of that locality [102–104]. More intense site use is indicated by abundant lithics and food refuse in the Grey Series at Taforalt, as well as an increased sedimentation rate due to wood ash contributions. Furthermore, the appearance of a cemetery in the Grey Series suggests that people had developed permanent ties to Taforalt by this period [18,105,106].

Similar associations of wet climate intervals with increased site occupation intensities have been observed archaeologically in other places and times among other hunter-gatherer cultures that relied on storable plant foods. Comparable phenomena were unfolding simultaneously in the Levant during GI-1, when Early Natufian foragers became increasingly sedentary at a time of increased primary productivity. Mobility was reduced to the point that Natufians invested resources into building permanent residential structures, and archaeofaunas reveal resource depression and exceptional diet broadening [107]. Another similar case study comes from the Ahmarian period of the northern Levant, where at the site of Üçağızlı I, a wetter interval identified isotopically by lower δ^13^C values among ungulates corresponds to higher occupation intensities as evidenced by diverse combustion features, dense lithic and faunal refuse, and a switch to logistical mobility strategies for the procurement of raw materials [108]. Rather than emphasizing storable plant foods, the occupants of Üçağızlı I engaged in meat smoking and drying.

At Taforalt and Rhafas, associations between climate amelioration and more sedentary land-use strategies likely arose from a combination of factors. First, mobility is expected to decline when a locale becomes more productive than the surrounding area [109]. Increased productivity of oaks and pines in montane regions during GI-1 would have provided local foragers better access to highly storable resources with a high caloric return rate relative to other plants and small game, particularly at Taforalt where evidence is stronger for processed acorn consumption coupled with long-duration and repeated stays during GI-1.

At the same time, there is a spike in dated LSA archaeological contexts in northwestern Africa during GI-1 (15–13 ka) which cannot easily be explained by a rearrangement of mobility strategies alone. It suggests a large-scale demographic transformation – potentially fueled by climate-driven increases in primary resource productivity on geographic scales beyond the spatial scope of this study. We propose that rising population densities and territorialities of neighboring groups would have restricted the mobility of hunter-gatherers at Taforalt and Rhafas, as has similarly been proposed for the Natufian [107,110]. Rising human population densities affected intensities of site occupations in the greater region, while at the same time and at finer geographic scales, increasingly productive patches of nut-bearing trees at high elevations would have intensified sedentism at some locales such as Taforalt.

Interestingly, preliminary evidence from Taforalt suggests that the GI-1 climate interval amplified an already existing trend towards increased local intensities of site occupations. Data are still limited, but site occupation intensities appear to increase in the brown/dark brown layer of Sectors 10 and 13 relative to earlier layers, including the Yellow Series which underlies the Grey Series in other parts of the site. If the brown layer dates to just prior to GI-1 during Heinrich Stadial 1, as the stable carbon isotope data from this study suggest, then human use of the site was increasing before climate amelioration took place. However, more work is required to assess the age and stratigraphic relationships of these layers. Even if occupation intensities were already on the rise due to technological or other reasons, we expect that the warm and moist climate of GI-1 caused regional primary productivity to increase. This productivity would have raised the carrying capacity of the environment, supporting accelerating human population sizes (sensu [111]), and likely also caused a threshold to be passed in the arboreal resource productivity of montane regions. The combination of these two factors operating simultaneously over different spatial scales resulted in a categorical change in forager land-use strategies.

Lastly, it is important to consider the δ^13^C isotope record as a whole, from the earliest MSA layers of Taforalt and Rhafas to the most recent LSA contexts. There may be a trend as observed in Taforalt Barbary sheep from lower δ^13^C values in MIS 5a-b to higher δ^13^C values in MIS 4 and again in Heinrich Stadial 1 (Fig 5). However, within this timeframe spanning more than 120 thousand years only the GI-1 climate interval produced a clearly measurable excursion in δ^13^C values within our dataset. We know from many other global, regional, and local climate records that the western Mediterranean experienced climate shifts during this time, many being just as extreme as GI-1 or more so [24]. The absence of this climatic variability in the carbon isotope proxy does not mean that northeastern Morocco was unaffected environmentally by climate changes. Rather, the GI-1 climate interval happened to coincide with a very different kind of archaeological context: an intense, rapidly accumulating occupation representing a very brief period. The data from earlier layers at Taforalt and Rhafas are more time-averaged because human presence on the landscape was light and sedimentation rates were relatively low, even during wetter climate intervals. Therefore, climate extremes may not be as well represented in paleoenvironmental proxies from the site. Additionally, gerbil (*Meriones* sp.) tooth δ^13^C values from Taforalt indicate that C4 plants often made up a small but measurable component of the local vegetation consumed by this taxon [112]. Even minor, variable contributions of C4 grass to herbivore diets, as well as fluctuating pCO_2_ and δ^13^C_atm_ values, have potential to complicate or obscure climate signals in stable carbon isotopes over long timescales [70,71]. The visibility of GI-1 forest expansion in the isotope record from Taforalt and Rhafas, together with the distinctive ensemble of human behaviors that accompany, is testament to important changes from the Middle to the Later Stone Age in population densities and the ways that human groups interacted with their landscapes.

## Conclusions

At Taforalt and Rhafas, Barbary sheep and gazelle δ^13^C values show a 1.5−2‰ decrease during the Greenland Interstadial 1/Bølling-Allerød climate interval. Given that equines and alcelaphines, which prefer to forage in open, grassy habitats do *not* experience a shift in δ^13^C values over this timeframe suggests that wooded montane and hilly environments were the areas principally affected by GI-1 climate change, at least locally. We argue that expansion of high-elevation forests and canopy in-filling took place near Taforalt and Rhafas during GI-1. The geographic scale of this phenomenon is not yet known, but it is similarly documented at Ifri El Baroud located 80 km west of Taforalt [12].

Forest expansion is linked closely to the initiation of the Grey Series at Taforalt, where marked changes in forager land-use behaviors are observed. These include intense exploitation of acorns and pine nuts with indirect evidence for plant processing and storage, dense concentrations of artifacts and food refuse despite extremely high rates of anthropogenic sedimentation, and the development of a cemetery at the back of the cave. These archaeological signals suggest more permanent ties to place [10]. Taken together, these features point to longer-duration site stays with a major activity being the exploitation of storable, forest-derived plant foods. Increased productivity of trees during GI-1 is expected to have contributed to or enabled the site’s intensive use during this time. In addition to local productivity, rising regional human population densities in GI-1, potentially linked to increases in primary productivity over large spatial scales, also likely contributed to increased human presence at Taforalt, Rhafas, and in Morocco more broadly.

There are preliminary indications that the suite of behavioral characteristics dating to GI-1 that differentiate the Grey Series of Taforalt from earlier layers may have arisen in a context of increasingly dense site occupations during the late stages of the cooler and drier Heinrich Stadial 1. Additional dating and paleoenvironmental analysis will constrain the age and timing of landscape evolution and categoric shifts in forager land-use over this important interval.

## Supporting information

S1 Table Contextual information, raw δ^13^C_enamel_ and δ^18^O_enamel_ data, and transformed δ^13^C_diet_ data for all sampled specimens, separated by taxon. (XLSX)

S2 Table δ^13^C_enamel_ and δ^18^O_enamel_ value summary statistics from sequentially sampled specimens. (XLSX)

S3 Table δ^13^C_enamel_ and δ^18^O_enamel_ values of pretreated and untreated samples. (XLSX)

S1 Fig δ^18^O_enamel_ values among Barbary sheep, gazelle, equines, and alcelaphines at Taforalt (left) and Rhafas (right), separated by temporal-stratigraphic interval with dates following Tables 1 and 2. (TIF)

S1 File Plots of intra-tooth δ^13^C_enamel_ (black) and δ^18^O_enamel_ (blue) values for each sequentially sampled specimen. (PDF)

## References

[1] Boisard S, Ben Arous E. A critical inventory and associated chronology of the Middle Stone Age and later stone age in Northwest Africa. J Open Archaeol Data. 2024;12. doi: 10.5334/joad.121

[2] Hogue J. The origin and development of the Pleistocene LSA in Northwest Africa: a case study from Grotte des Pigeons (Taforalt), Morocco. University of Oxford; 2014.

[3] Sari L. Diachronic variation in microlith production systems during the late Pleistocene, Algeria. In: Sari L, Mutri G, editors. Variability of late Pleistocene and Holocene microlithic industries in Northern and Eastern Africa. Springer; 2022.

[4] Desmond A, Barton N, Bouzouggar A, Douka K, Fernandez P, Humphrey L. ZooMS identification of bone tools from the North African Later Stone Age. J Archaeol Sci. 2018;98:149–57.

[5] Desmond A. A multi-method approach to understanding prehistoric bone tools. University of Oxford; 2022.

[6] Desmond A. Bone tool proxy evidence for coiled basketry production in the North African Palaeolithic. J African Arch. 2022;20(2):156–75. doi: 10.1163/21915784-bja10018

[7] Hachi S, Fröhlich F, Gendron-Badou A, de Lumley H, Roubet C, Abdessadok S. Figurines du Paléolithique supérieur en matière minérale plastique cuite d’Afalou Bou Rhummel (Babors, Algérie). Premières analyses par spectroscopie d’absorption Infrarouge. Anthropologie. 2002;106(1):57–97.

[8] Sari L. Hat Rockshelter, Algeria. In: Beyin A, Wright DK, Wilkins J, Olszewski DI, editors. Handbook of Pleistocene Archaeology of Africa. Springer; 2023.

[9] Barton RNE, Bouzouggar A, Hogue JT, Lee S, Collcutt SN, Ditchfield P. Origins of the iberomaurusian in NW Africa: new AMS radiocarbon dating of the Middle and Later Stone Age deposits at Taforalt Cave, Morocco. J Hum Evol. 2013;65(3):266–81. doi: 10.1016/j.jhevol.2013.06.003 23891007

[10] Barton RNE, Bouzouggar A, Collcutt SN, Humphrey LT. Cemeteries and sedentism in the Later Stone Age of NW Africa: excavations at Grotte des Pigeons, Taforalt, Morocco. Römisch-Germanischen Zentralmuseums; 2019.

[11] Nami M, Moser J. La grotte d’Ifri n’Ammar; tome 2: Le paléolithique moyen. Wiesbaden: Reichert; 2010.

[12] Potì A, Kehl M, Broich M, Marco YC, Hutterer R, Jentke T. Human occupation and environmental change in the western Maghreb during the Last Glacial Maximum (LGM) and the Late Glacial. New evidence from the Iberomaurusian site Ifri El Baroud (northeast Morocco). Quat Sci Rev. 2019;220:87–110.

[13] Humphrey LT, Freyne A, Berridge P, Berridge P. Human burial evidence. In: Barton RNE, Bouzouggar A, Collcutt SN, Humphrey LT, editors. Cemeteries and sedentism in the Later Stone Age of NW Africa: excavations at Grotte des Pigeons, Taforalt, Morocco. Römisch-Germanischen Zentralmuseums; 2019: 443–82.

[14] Humphrey L, Freyne A, Bouzouggar A. Mind the gap: funerary behavior during the Iberomaurusian. In: Gaudzinski-Windheuser S, Jöris O, editors. The beef behind all possible pasts: the tandem festschrift in honour of Elaine Turner and Martin Street. RGZM; 2021: 459–70.

[15] Carrión Marco Y, Morales J, Portillo M, Pérez-Jordà G, Peña-Chocarro L, Zapata L. The use of wild plants in the Palaeolithic and Neolithic of Northwestern Africa: preliminary results from the PALEOPLANT project. In: Mercuri A, D’Andrea A, Fornaciari R, Höhn A, editors. Cham: Springer; 2018.

[16] Morales J. The contribution of botanical macro-remains to the study of wild plant consumption during the Later Stone Age and the Neolithic of North-Western Africa. J Archaeol Sci Rep. 2018;22:401–12.

[17] Moubtahij Z, McCormack J, Bourgon N, Trost M, Sinet-Mathiot V, Fuller BT. Isotopic evidence of high reliance on plant food among Later Stone Age hunter-gatherers at Taforalt, Morocco. Nat Ecol Evol. 2024:1–11. 38684738 10.1038/s41559-024-02382-z PMC11090808

[18] Humphrey LT, De Groote I, Morales J, Barton N, Collcutt S, Bronk Ramsey C, et al. Earliest evidence for caries and exploitation of starchy plant foods in Pleistocene hunter-gatherers from Morocco. Proc Natl Acad Sci USA. 2014;111(3):954–9. doi: 10.1073/pnas.1318176111 24395774 PMC3903197

[19] Klasen N, Kehl M, Mikdad A, Brückner H, Weniger GC. Chronology and formation processes of the Middle to Upper Palaeolithic deposits of Ifri n’Ammar using multi-method luminescence dating and micromorphology. Quat Int. 2018;485:89–102.

[20] Taylor VK, Barton RNE, Bell M, Bouzouggar A, Collcutt S, Black S. The Epipalaeolithic (Iberomaurusian) at Grotte des Pigeons (Taforalt), Morocco: a preliminary study of the land Mollusca. Quat Int. 2011;244(1):5–14.

[21] Campmas E, Chakroun A, Merzoug S. Preliminary data on the exploitation of marine malacofauna by the Iberomaurusian groups of the Abri Alain rock shelter (Oran, Algeria). PALEO Revue d’archéologie préhistorique. 2016;(27):83–104.

[22] Barker G, Bennett P, Farr L, Hill E, Hunt C, Lucarini G. The Cyrenaican Prehistory Project 2012: the fifth season of investigations of the Haua Fteah cave. Libyan Studies. 2012;43:115–36.

[23] Cacho I, Grimalt JO, Canals M, Sbaffi L, Shackleton NJ, Schönfeld J, et al. Variability of the western Mediterranean Sea surface temperature during the last 25,000 years and its connection with the Northern Hemisphere climatic changes. Paleoceanography. 2001;16(1):40–52. doi: 10.1029/2000pa000502

[24] Martrat B, Grimalt JO, Lopez-Martinez C, Cacho I, Sierro FJ, Flores JA, et al. Abrupt temperature changes in the Western Mediterranean over the past 250,000 years. Science. 2004;306(5702):1762–5. doi: 10.1126/science.1101706 15576615

[25] Combourieu Nebout N, Peyron O, Dormoy I, Desprat S, Beaudouin C, Kotthoff U, et al. Rapid climatic variability in the west Mediterranean during the last 25 000 years from high resolution pollen data. Clim Past. 2009;5(3):503–21. doi: 10.5194/cp-5-503-2009

[26] García-Alix A, Jiménez-Moreno G, Jiménez-Espejo FJ, García-García F, Huertas AD. An environmental snapshot of the Bølling interstadial in Southern Iberia. Quat Res. 2014;81(2):284–94.

[27] Genty D, Blamart D, Ghaleb B, Plagnes V, Causse C, Bakalowicz M. Timing and dynamics of the last deglaciation from European and North African δ^13^C stalagmite profiles—comparison with Chinese and South Hemisphere stalagmites. Quat Sci Rev. 2006;25(17–18):2118–42.

[28] Rhoujjati A, Cheddadi R, Taieb M, Baali A, Ortu E. Environmental changes over the past c. 29,000 years in the Middle Atlas (Morocco): a record from Lake Ifrah. J Arid Environ. 2010;74(7):737–45.

[29] El Bait MN, Rhoujjati A, Eynaud F, Benkaddour A, Dezileau L, Wainer K. An 18,000‐year pollen and sedimentary record from the cedar forests of the Middle Atlas, Morocco. J Quat Sci. 2014;29(5):423–32.

[30] Tabel J, Khater C, Rhoujjati A, Dezileau L, Bouimetarhan I, Carre M, et al. Environmental changes over the past 25,000 years in the southern Middle Atlas, Morocco. J Quat Sci. 2016;31(2):93–102.

[31] Abirham T, Bekele A, Yihune M. Population status, distribution and seasonal range of Grevy’s zebra (*Equus grevyi*) in a protected savannah area. Afr Zool. 2023;58(3–4):59–66.

[32] Etchart JL. Evaluating water use and seasonal ranges of desert Bighorn sheep and Aoudad in the Sierra Vieja mountains, Texas. M.Sc. Thesis. Sul Ross State University; 2021.

[33] Gebre B, Yirga S. Seasonal home range of Swayne’s Hartebeest (*Alcelaphus buselaphus swaynei*) in Senkele Swayne’s Hartebeest Sanctuary. SINET. 2004;27(2):121–6.

[34] Githiru M. The forgotten Grevy’s zebra *Equus grevyi* population along the Kasigau Corridor ranches, SE Kenya: recent records and conservation issues. Afr J Ecol. 2017;55(4):554–63.

[35] Hampy DB. Home range and seasonal movement of Barbary sheep in the Palo Duro Canyon. Texas Tech University; 1978.

[36] Klingel H. The social organisation and population ecology of the plains zebra (*Equus quagga*). Afr Zool. 1969;4(2).

[37] Owen‐Smith N, Hopcraft G, Morrison T, Chamaillé‐Jammes S, Hetem R, Bennitt E. Movement ecology of large herbivores in African savannas: current knowledge and gaps. Mamm Rev. 2020;50(3):252–66.

[38] Tesfai RT, Parrini F, Owen-Smith N, Moehlman PD. How spatial and dietary overlap with domestic livestock affect African wild ass nutrition on the Messir Plateau (Eritrea). J Mammal. 2021;102(4):1174–85.

[39] Doerschner N, Fitzsimmons KE, Ditchfield P, McLaren SJ, Steele TE, Zielhofer C, et al. A new chronology for Rhafas, Northeast Morocco, spanning the North African Middle Stone Age through to the Neolithic. PLoS One. 2016;11(9):e0162280. doi: 10.1371/journal.pone.0162280 27654350 PMC5031315

[40] Ryan WBF, Carbotte SM, Coplan JO, O’Hara S, Melkonian A, Arko R, et al. Global multi‐resolution topography synthesis. Geochem Geophys Geosyst. 2009;10(3). doi: 10.1029/2008gc002332

[41] Roche J. Note préliminaire sur les fouilles de la grotte de Taforalt (Maroc Oriental). Hespéris. 1953;40:89–116.

[42] Roche J. L’epipaléolithique marocaine. Lisbon: Fondation Calouste Gulbenkian; 1963.

[43] Roche J. L’aterian de la grotte de Taforalt (Maroc oriental). Bull Archeol Marocaine. 1967;7:11–56.

[44] Roche J. Les industries paléolithiques de la grotte de Taforalt (Maroc oriental). Quaternaria. 1969;11:89–100.

[45] Roche J. Cadre chronologique de l’Epipaléolithique marocain. In: Actes du IXè Congrès de l’UISPP: Chronologie et Synchronisme dans la Préhistoire Circum-Méditerranéenne. 1976: 153–167.

[46] Raynal JP. Mission préhistorique et paléontologique française au Taforalt Maroc: rapport d’activité pour l’année. Bull. Archéol. Marocaine. 1980;12: 69–71.

[47] Bouzouggar A, Barton N, Vanhaeren M, d’Errico F, Collcutt S, Higham T. 82,000 year-old shell beads from North Africa and implications for the origins of modern human behavior. Proc Natl Acad Sci. 2007;104:9964–9. 17548808 10.1073/pnas.0703877104 PMC1891266

[48] Bouzouggar A, Barton RNE, Blockley S, Bronk-Ramsey C, Collcutt SN, Gale R. Reevaluating the age of the Iberomaurusian in Morocco. Afr Archaeol Rev. 2008;25:3–19.

[49] Collcutt SN. Lithostratigraphies and sediments. In: Barton RNE, Bouzouggar A, Collcutt SN, Humphrey LT, editors. Cemeteries and Sedentism in the Later Stone Age of NW Africa: Excavations at Grotte des Pigeons, Taforalt, Morocco. Römisch-Germanischen Zentralmuseums; 2019: 239–308.

[50] Staff RA, Ditchfield P, Rhodes E, Schwenninger JL, Clark-Balzan L, Lee S, et al. Chronology. In: Barton RNE, Bouzouggar A, Collcutt SN, Humphrey LT, editors. Cemeteries and Sedentism in the Later Stone Age of NW Africa: Excavations at Grotte des Pigeons, Taforalt, Morocco. Römisch-Germanischen Zentralmuseums; 2019: 239–308.

[51] Uzunidis A, Fernandez P, Bouzouggar A, Barton N, Humphrey L, Kuhn S. Herbivore dental wear analysis since the end of the middle Pleistocene to the beginning of the Holocene in different archaeological contexts of Morocco (Bizmoune, El Khenzira and Taforalt). Paleo. 2023:208–27. doi: 10.4000/paleo.8411

[52] Barton RNE, Lane CS, Albert PG, White D, Collcutt SN, Bouzouggar A. The role of cryptotephra in refining the chronology of Late Pleistocene human evolution and cultural change in North Africa. Quat Sci Rev. 2015;118:151–69.

[53] Humphrey L, Bello SM, Turner E, Bouzouggar A, Barton N. Iberomaurusian funerary behaviour: evidence from Grotte des Pigeons, Taforalt, Morocco. J Hum Evol. 2012;62(2):261–73. doi: 10.1016/j.jhevol.2011.11.003 22154088

[54] Humphrey L, Freyne A, van de Loosdrecht M, Hogue JT, Turner E, Barton N, et al. Infant funerary behavior and kinship in Pleistocene hunter-gatherers from Morocco. J Hum Evol. 2019;135:102637. doi: 10.1016/j.jhevol.2019.07.001 31421318

[55] Clark-Balzan LA, Candy I, Schwenninger JL, Bouzouggar A, Blockley S, Nathan R. Coupled U-series and OSL dating of a Late Pleistocene cave sediment sequence, Morocco, North Africa: significance for constructing Palaeolithic chronologies. Quat Geochronol. 2012;12:53–64.

[56] Wengler L. Cultures préhistoriques et formations quaternaires au Maroc oriental. Relations entre comportements et paléoenvironments au Paléolithique moyen. Université de Bordeaux I; 1993.

[57] Cassinello J, Acevedo P, Hortal J. Prospects for population expansion of the exotic aoudad (Ammotragus lervia; Bovidae) in the Iberian Peninsula: clues from habitat suitability modelling. Divers Distrib. 2006;12(6):666–78.

[58] Acevedo P, Cassinello J, Hortal J, Gortázar C. Invasive exotic aoudad (*Ammotragus lervia*) as a major threat to native Iberian ibex (*Capra pyrenaica*): a habitat suitability model approach. Divers Distrib. 2007;13(5):587–97.

[59] Loggers CO, Thévenot M, Aulagnier S. Status and distribution of Moroccan wild ungulates. Biol Conserv. 1992;59(1):9–18.

[60] Cuzin F. Les grands mammifères du Maroc méridional (Haut Atlas, Anti Atlas et Sahara): distribution, écologie et conservation. Université Montpellier II; 2003.

[61] Cerling TE, Wang Y, Quade J. Expansion of C4 ecosystems as an indicator of global ecological change in the late Miocene. Nature. 1993;361(6410):344–5. doi: 10.1038/361344a0

[62] Kohn MJ. Carbon isotope compositions of terrestrial C3 plants as indicators of (paleo)ecology and (paleo)climate. Proc Natl Acad Sci U S A. 2010;107(46):19691–5. doi: 10.1073/pnas.1004933107 21041671 PMC2993332

[63] Winslow JC, Hunt ER, Piper SC. The influence of seasonal water availability on global C3 versus C4 grassland biomass and its implications for climate change research. Ecol Modell. 2003;163(1–2):153–73.

[64] Murphy BP, Bowman DM. Seasonal water availability predicts the relative abundance of C3 and C4 grasses in Australia. Glob Ecol Biogeogr. 2007;16(2):160–9.

[65] Edwards EJ, Still CJ. Climate, phylogeny and the ecological distribution of C4 grasses. Ecol Lett. 2008;11(3):266–76. doi: 10.1111/j.1461-0248.2007.01144.x 18201200

[66] Vogel JC. Isotopic assessment of the dietary habits of ungulates. S Afr J Sci. 1978;74(8):298.

[67] Arens NC, Jahren AH, Amundson R. Can C3 plants faithfully record the carbon isotopic composition of atmospheric carbon dioxide? Paleobiology. 2000;26(1):137–64. doi: 10.1666/0094-8373(2000)026<0137:ccpfrt>2.0.co;2

[68] Schubert BA, Jahren AH. The effect of atmospheric CO2 concentration on carbon isotope fractionation in C3 land plants. Geochimica et Cosmochimica Acta. 2012;96:29–43. doi: 10.1016/j.gca.2012.08.003

[69] Hartman G, Danin A. Isotopic values of plants in relation to water availability in the Eastern Mediterranean region. Oecologia. 2010;162(4):837–52. doi: 10.1007/s00442-009-1514-7 19956974 PMC2841277

[70] Wang G, Li J, Liu X, Li X. Variations in carbon isotope ratios of plants across a temperature gradient along the 400 mm isoline of mean annual precipitation in north China and their relevance to paleovegetation reconstruction. Quat Sci Rev. 2013;63:83–90.

[71] Feranec RS. Stable carbon isotope values reveal evidence of resource partitioning among ungulates from modern C3-dominated ecosystems in North America. Palaeogeogr Palaeoclimatol Palaeoecol. 2007;252(3–4):575–85. doi: 10.1016/j.palaeo.2007.05.012

[72] Bonafini M, Pellegrini M, Ditchfield P, Pollard AM. Investigation of the ‘canopy effect’ in the isotope ecology of temperate woodlands. J Archaeol Sci. 2013;40(11):3926–35.

[73] van der Merwe NJ, Medina E. The canopy effect, carbon isotope ratios and foodwebs in Amazonia. J Archaeol Sci. 1991;18(3):249–59.

[74] Cerling TE, Hart JA, Hart TB. Stable isotope ecology in the Ituri Forest. Oecologia. 2004;138(1):5–12. doi: 10.1007/s00442-003-1375-4 14530961

[75] Fuller BT, Fuller JL, Harris DA, Hedges REM. Detection of breastfeeding and weaning in modern human infants with carbon and nitrogen stable isotope ratios. Am J Phys Anthropol. 2006;129(2):279–93. doi: 10.1002/ajpa.20249 16261548

[76] Orr AJ, Newsome SD, Laake JL, VanBlaricom GR, DeLong RL. Ontogenetic dietary information of the California sea lion (*Zalophus californianus*) assessed using stable isotope analysis. Mar Mamm Sci. 2012;28(4):714–32.

[77] Polischuk SC, Hobson KA, Ramsay MA. Use of stable-carbon and nitrogen isotopes to assess weaning and fasting in female polar bears and their cubs. Can J Zool. 2001;79(3):499–511.

[78] Jenkins SG, Partridge ST, Stephenson TR, Farley SD, Robbins CT. Nitrogen and carbon isotope fractionation between mothers, neonates, and nursing offspring. Oecologia. 2001;129(3):336–41. doi: 10.1007/s004420100755 28547188

[79] Tsutaya T, Yoneda M. Reconstruction of breastfeeding and weaning practices using stable isotope and trace element analyses: a review. Am J Phys Anthropol. 2015;156(Suppl 59):2–21. doi: 10.1002/ajpa.22657 25407359

[80] Crowell-Davis SL, Houpt KA, Carnevale J. Feeding and drinking behavior of mares and foals with free access to pasture and water. J Anim Sci. 1985;60(4):883–9. doi: 10.2527/jas1985.604883x 3988655

[81] McKinney T, Smith TW. Diets of adults and lambs of desert bighorn sheep during years of varying rainfall in central Arizona. Southwest Nat. 2007;52(4):520–7.

[82] Balasse M. Reconstructing dietary and environmental history from enamel isotopic analysis: time resolution of intra‐tooth sequential sampling. Int J Osteoarchaeol. 2002;12(3):155–65.

[83] Balasse M, Obein G, Ughetto‐Monfrin J, Mainland I. Investigating seasonality and season of birth in past herds: a reference set of sheep enamel stable oxygen isotope ratios. Archaeometry. 2011;54(2):349–68. doi: 10.1111/j.1475-4754.2011.00624.x

[84] Spencer F, Verostick K, Serna A, Stantis C, Bowen GJ. Effects of particle size, storage conditions, and chemical pretreatments on carbon and oxygen isotopic measurements of modern tooth enamel. Sci Justice. 2024;64(2):193–201. doi: 10.1016/j.scijus.2024.01.004 38431376

[85] Varkuleviciute K, Gron KJ, Patterson WP, Panelli C, Rossi S, Timsic S. Transhumance in the Early Neolithic? Carbon and oxygen isotope insights into sheep husbandry at Arene Candide, Northern Italy. J Archaeol Sci Rep. 2021;40:103240.

[86] Cerling TE, Bernasconi SM, Hofstetter LS, Jaggi M, Wyss F, Rudolf von Rohr C. CH4/CO2 ratios and carbon isotope enrichment between diet and breath in herbivorous mammals. Front Ecol Evolution. 2021;9:638568.

[87] Turner E. Large Mammalian Fauna. In: Barton RNE, Bouzouggar A, Collcutt SN, Humphrey LT, editors. Cemeteries and Sedentism in the Later Stone Age of NW Africa: Excavations at Grotte des Pigeons, Taforalt, Morocco. Römisch-Germanischen Zentralmuseums; 2019: 239–308.

[88] Segura A, Moreno E. Foraging habitat use by sympatric Cuvier’s Gazelle, Dama Gazelle, and Dorcas Gazelle on a private reserve in Morocco. J Mammal. 2024;105(6):1345–52. doi: 10.1093/jmammal/gyae079

[89] Brooks JR, Flanagan LB, Buchmann N, Ehleringer JR. Carbon isotope composition of boreal plants: functional grouping of life forms. Oecologia. 1997;110(3):301–11. doi: 10.1007/s004420050163 28307218

[90] Kelly CK, Woodward FI. Ecological correlates of carbon isotope composition of leaves: a comparative analysis testing for the effects of temperature, CO2 and O2 partial pressures and taxonomic relatedness on δ^13^C. J Ecol. 1995:509–15.

[91] Weiguo L, Xiahong F, Youfeng N, Qingle Z, Yunning C, Zhisheng AN. δ^13^C variation of C3 and C4 plants across an Asian monsoon rainfall gradient in arid northwestern China. Glob Chang Biol. 2005;11(7):1094–100.

[92] Pate FD, Krull E. Carbon isotope discrimination by C3 pasture grasses along a rainfall gradient in South Australia: implications for palaeoecological studies. Quaternary Australasia. 2007;14:29–33.

[93] Murphy BP, Bowman DM. The carbon and nitrogen isotope composition of Australian grasses in relation to climate. Funct Ecol. 2009;23(6):1040–9.

[94] Körner C, Farquhar GD, Roksandic Z. A global survey of carbon isotope discrimination in plants from high altitude. Oecologia. 1988;74(4):623–32. doi: 10.1007/BF00380063 28311772

[95] Liu X, Zhao L, Gasaw M, Gao D, Qin D, Ren J. Foliar δ^13^C and δ^15^N values of C3 plants in the Ethiopia Rift Valley and their environmental controls. Chin Sci Bull. 2007;52:1265–73.

[96] Wengler L, Vernet J-L. Vegetation, sedimentary deposits and climates during the Late Pleistocene and Holocene in eastern Morocco. Palaeogeogr Palaeoclimatol Palaeoecol. 1992;94(1–4):141–67. doi: 10.1016/0031-0182(92)90117-n

[97] Nardini A, Salleo S, Lo Gullo MA, Pitt F. Different responses to drought and freeze stress of Quercus ilex L. growing along a latitudinal gradient. Plant Ecol. 2000;148(2):139–47. doi: 10.1023/a:1009840203569

[98] Reade H, O’Connell TC, Barker G, Stevens RE. Pleistocene and Holocene palaeoclimates in the Gebel Akhdar (Libya) estimated using herbivore tooth enamel oxygen isotope compositions. Quat Int. 2016;404:150–62.

[99] Binford LR. Willow smoke and dogs’ tails: hunter-gatherer settlement systems and archaeological site formation. Am Antiq. 1980;45(1):4–20.

[100] Rowley-Conwy P, Zvelebil M. Saving it for later: storage by prehistoric hunter-gatherers in Europe. In: Halstead P, O’Shea J, editors. Bad year economics: cultural responses to risk and uncertainty. Cambridge University Press; 1989: 40–56.

[101] Tushingham S, Bettinger RL. Why foragers choose acorns before salmon: Storage, mobility, and risk in aboriginal California. J Anthropol Archaeol. 2013;32(4):527–37.

[102] Hofman JL. Hunter-gatherer mortuary variability: toward an explanatory model. University of Tennessee; 1986.

[103] Littleton J, Allen H. Hunter-gatherer burials and the creation of persistent places in southeastern Australia. J Anthropol Archaeol. 2007;26(2):283–98.

[104] Munro N. Zooarchaeological measures of hunting pressure and occupation intensity in the Natufian: implications for agricultural origins. Curr Anthropol. 2004;45(S4):S5–34.

[105] Worthey KB, Stiner MC, Quade J, Rowland JC, Açıkkol A, Baykara I, et al. Paleolithic Human Responses to Changing Aridity at Üçağızlı I cave, southern-coastal Turkey: Application of a Novel Carbon Isotope-Based Method. J Archaeol Method Theory. 2022;29(4):1190–228. doi: 10.1007/s10816-022-09553-x

[106] Kelly RL. The lifeways of hunter-gatherers: the foraging spectrum. Cambridge University Press; 2013.

[107] Stiner M, Munro N, Surovell T, Tchernov E, Bar-Yosef O. Paleolithic population growth pulses evidenced by small animal exploitation. Science. 1999;283(5399):190–4. doi: 10.1126/science.283.5399.190 9880245

[108] Richerson PJ, Boyd R, Bettinger RL. Was agriculture impossible during the Pleistocene but mandatory during the Holocene? A climate change hypothesis. Am Antiq. 2001;66(3):387–411.

[109] Barton RNE, Bouzouggar A, Collcutt SN, Marco YC, Clark-Balzan L, Debenham NC. Reconsidering the MSA to LSA transition at Taforalt Cave (Morocco) in the light of new multi-proxy dating evidence. Quat Int. 2016;413:36–49.

[110] Wengler L. La transition du Moustérien à l’Atérien. Anthropologie. 1997;101(3):448–81.

[111] Mercier N, Wengler L, Valladas H, Joron JL, Froget L, Reyss JL. The Rhafas Cave (Morocco): chronology of the Mousterian and Aterian archaeological occupations and their implications for Quaternary geochronology based on luminescence (TL/OSL) age determinations. Quat Geochronol. 2007;2(1–4):309–13.

[112] Jeffrey A. Exploring palaeoaridity using stable oxygen and carbon isotopes in small mammal teeth: a case study from two Late Pleistocene archaeological cave sites in Morocco, North Africa. Doctoral dissertation. University of Oxford; 2016.

