# Correction: Expansion of forest cover and coeval shifts in Later Stone Age land-use at Taforalt and Rhafas Caves, Morocco, as inferred from carbon isotopes in ungulate tooth enamel

**DOI:** 10.1371/journal.pone.0336238

**Published:** 2025-11-04

**Authors:** Kayla B. Worthey, Philippe Fernandez, Elaine Turner, Teresa E. Steele, Louise Humphrey, R. Nick E. Barton, Jean-Jacques Hublin, Abdeljalil Bouzouggar

In Table 1, the reference list in the fifth column are incorrect. Please see the correct Table 1 here.

The reference cited in Fig 1, “Locations of Taforalt and Rhafas caves in northeastern Morocco.” is incorrect. Please see the complete, correct Fig 1 caption here.

The reference cited in Fig 2, “Taforalt Sector 8 stratigraphy of the Grey Series and Yellow Series.” is incorrect. Please see the complete, correct Fig 2 caption here.

There is an error in reference 40. The correct reference is: Roche J. Note préliminaire sur les fouilles de la grotte de Taforalt (Maroc Oriental). Hespéris. 1953;40:89–116.

There is an error in reference 41. The correct reference is: Roche J. L’epipaléolithique marocaine. Lisbon: Fondation Calouste Gulbenkian; 1963.

There is an error in reference 42. The correct reference is: Roche J. L’aterian de la grotte de Taforalt (Maroc oriental). Bull Archeol Marocaine. 1967;7:11–56.

There is an error in reference 43. The correct reference is: Roche J. Les industries paléolithiques de la grotte de Taforalt (Maroc oriental). Quaternaria. 1969;11:89–100.

There is an error in reference 44. The correct reference is: Roche J. Cadre chronologique de l’Epipaléolithique marocain. In: Actes du IXè Congrès de l’UISPP: Chronologie et Synchronisme dans la Préhistoire Circum-Méditerranéenne. 1976: 153–167.

There is an error in reference 45. The correct reference is: Raynal JP. Mission préhistorique et paléontologique française au Taforalt Maroc: rapport d’activité pour l’année. Bull. Archéol. Marocaine. 1980;12: 69–71.

There is an error in reference 46. The correct reference is: Bouzouggar A, Barton N, Vanhaeren M, d’Errico F, Collcutt S, Higham T. 82,000 year-old shell beads from North Africa and implications for the origins of modern human behavior. Proc Natl Acad Sci. 2007;104:9964–9.

There is an error in reference 47. The correct reference is: Bouzouggar A, Barton RNE, Blockley S, Bronk-Ramsey C, Collcutt SN, Gale R. Reevaluating the age of the Iberomaurusian in Morocco. Afr Archaeol Rev. 2008;25:3–19.

There is an error in reference 48. The correct reference is: Barton RNE, Bouzouggar A, Collcutt SN, Marco YC, Clark-Balzan L, Debenham NC. Reconsidering the MSA to LSA transition at Taforalt Cave (Morocco) in the light of new multi-proxy dating evidence. Quat Int. 2016;413:36–49.

There is an error in reference 49. The correct reference is: Wengler L. Cultures préhistoriques et formations quaternaires au Maroc oriental. Relations entre comportements et paléoenvironments au Paléolithique moyen. Université de Bordeaux I; 1993.

There is an error in reference 50. The correct reference is: Wengler L. La transition du Moustérien à l’Atérien. Anthropologie. 1997;101(3):448–81.

There is an error in reference 51. The correct reference is: Mercier N, Wengler L, Valladas H, Joron JL, Froget L, Reyss JL. The Rhafas Cave (Morocco): chronology of the Mousterian and Aterian archaeological occupations and their implications for Quaternary geochronology based on luminescence (TL/OSL) age determinations. Quat Geochronol. 2007;2(123–4):309–13.

There is an error in reference 52. The correct reference is: Ryan WBF, Carbotte SM, Coplan JO, O’Hara S, Melkonian A, Arko R, et al. Global multi‐resolution topography synthesis. Geochem Geophys Geosyst. 2009;10(3). https://doi.org/10.1029/2008gc002332

There is an error in reference 55. The correct reference is: Uzunidis A, Fernandez P, Bouzouggar A, Barton N, Humphrey L, Kuhn S. Herbivore dental wear analysis since the end of the middle Pleistocene to the beginning of the Holocene in different archaeological contexts of Morocco (Bizmoune, El Khenzira and Taforalt). Paleo. 2023:208–27. https://doi.org/10.4000/paleo.8411

There is an error in reference 56. The correct reference is: Collcutt SN. Lithostratigraphies and sediments. In: Barton RNE, Bouzouggar A, Collcutt SN, Humphrey LT, editors. Cemeteries and Sedentism in the Later Stone Age of NW Africa: Excavations at Grotte des Pigeons, Taforalt, Morocco. Römisch-Germanischen Zentralmuseums; 2019:239–308.

There is an error in reference 57. The correct reference is: Clark-Balzan LA, Candy I, Schwenninger JL, Bouzouggar A, Blockley S, Nathan R. Coupled U-series and OSL dating of a Late Pleistocene cave sediment sequence, Morocco, North Africa: significance for constructing Palaeolithic chronologies. Quat Geochronol. 2012;12:53–64.

There is an error in reference 58. The correct reference is: Staff RA, Ditchfield P, Rhodes E, Schwenninger JL, Clark-Balzan L, Lee S, et al. Chronology. In: Barton RNE, Bouzouggar A, Collcutt SN, Humphrey LT, editors. Cemeteries and Sedentism in the Later Stone Age of NW Africa: Excavations at Grotte des Pigeons, Taforalt, Morocco. Römisch-Germanischen Zentralmuseums; 2019: 239–308.

There is an error in reference 59. The correct reference is: Barton RNE, Lane CS, Albert PG, White D, Collcutt SN, Bouzouggar A. The role of cryptotephra in refining the chronology of Late Pleistocene human evolution and cultural change in North Africa. Quat Sci Rev. 2015;118:151–69.

There is an error in reference 60. The correct reference is: Cassinello J, Acevedo P, Hortal J. Prospects for population expansion of the exotic aoudad (Ammotragus lervia; Bovidae) in the Iberian Peninsula: clues from habitat suitability modelling. Divers Distrib. 2006;12(6):666–78.

There is an error in reference 61. The correct reference is: Acevedo P, Cassinello J, Hortal J, Gortázar C. Invasive exotic aoudad (Ammotragus lervia) as a major threat to native Iberian ibex (Capra pyrenaica): a habitat suitability model approach. Divers Distrib. 2007;13(5):587–97.

There is an error in reference 62. The correct reference is: Loggers CO, Thévenot M, Aulagnier S. Status and distribution of Moroccan wild ungulates. Biol Conserv. 1992;59(1):9–18.

There is an error in reference 63. The correct reference is: Cuzin F. Les grands mammifères du Maroc méridional (Haut Atlas, Anti Atlas et Sahara): distribution, écologie et conservation. Université Montpellier II; 2003.

There is an error in reference 64. The correct reference is: Cerling TE, Wang Y, Quade J. Expansion of C4 ecosystems as an indicator of global ecological change in the late Miocene. Nature. 1993;361(6410):344–5. https://doi.org/10.1038/361344a0

There is an error in reference 65. The correct reference is: Kohn MJ. Carbon isotope compositions of terrestrial C3 plants as indicators of (paleo)ecology and (paleo)climate. Proc Natl Acad Sci U S A. 2010;107(46):19691–5. https://doi.org/10.1073/pnas.1004933107 PMID: 21041671

There is an error in reference 66. The correct reference is: Winslow JC, Hunt ER, Piper SC. The influence of seasonal water availability on global C3 versus C4 grassland biomass and its implications for climate change research. Ecol Modell. 2003;163(1–2):153–73

There is an error in reference 67. The correct reference is: Murphy BP, Bowman DM. Seasonal water availability predicts the relative abundance of C3 and C4 grasses in Australia. Glob Ecol Biogeogr. 2007;16(2):160–9.

There is an error in reference 68. The correct reference is: Edwards EJ, Still CJ. Climate, phylogeny and the ecological distribution of C4 grasses. Ecol Lett. 2008;11(3):266–76. https://doi.org/10.1111/j.1461- 0248.2007.01144.x PMID: 18201200

There is an error in reference 69. The correct reference is: Vogel JC. Isotopic assessment of the dietary habits of ungulates. S Afr J Sci. 1978;74(8):298.

There is an error in reference 70. The correct reference is: Arens NC, Jahren AH, Amundson R. Can C3 plants faithfully record the carbon isotopic composition of atmospheric carbon dioxide? Paleobiology. 2000;26(1):137–64. https://doi.org/10.1666/0094-8373(2000)0262.0.co;2

There is an error in reference 71. The correct reference is: Schubert BA, Jahren AH. The effect of atmospheric CO2 concentration on carbon isotope fractionation in C3 land plants. Geochimica et Cosmochimica Acta. 2012;96:29–43. https://doi.org/10.1016/j.gca.2012.08.003

There is an error in reference 72. The correct reference is: Hartman G, Danin A. Isotopic values of plants in relation to water availability in the Eastern Mediterranean region. Oecologia. 2010;162(4):837–52. https://doi.org/10.1007/s00442-009-1514-7 PMID: 19956974

There is an error in reference 73. The correct reference is: Wang G, Li J, Liu X, Li X. Variations in carbon isotope ratios of plants across a temperature gradient along the 400 mm isoline of mean annual precipitation in north China and their relevance to paleovegetation reconstruction. Quat Sci Rev. 2013;63:83–90.

There is an error in reference 73. The correct reference is: Feranec RS. Stable carbon isotope values reveal evidence of resource partitioning among ungulates from modern C3-dominated ecosystems in North America. Palaeogeogr Palaeoclimatol Palaeoecol. 2007;252(3–4):575–85. https://doi.org/10.1016/j.palaeo.2007.05.012

There is an error in reference 74. The correct reference is: Bonafini M, Pellegrini M, Ditchfield P, Pollard AM. Investigation of the ‘canopy effect’ in the isotope ecology of temperate woodlands. J Archaeol Sci. 2013;40(11):3926–35.

There is an error in reference 75. The correct reference is: van der Merwe NJ, Medina E. The canopy effect, carbon isotope ratios and foodwebs in Amazonia. J Archaeol Sci. 1991;18(3):249–59.

There is an error in reference 76. The correct reference is: Cerling TE, Hart JA, Hart TB. Stable isotope ecology in the Ituri Forest. Oecologia. 2004;138(1):5–12. https://doi.org/10.1007/s00442-003-1375-4 PMID: 14530961

There is an error in reference 77. The correct reference is: Fuller BT, Fuller JL, Harris DA, Hedges REM. Detection of breastfeeding and weaning in modern human infants with carbon and nitrogen stable isotope ratios. Am J Phys Anthropol. 2006;129(2):279–93. https://doi.org/10.1002/ajpa.20249 PMID: 16261548.

There is an error in reference 78. The correct reference is: Orr AJ, Newsome SD, Laake JL, VanBlaricom GR, DeLong RL. Ontogenetic dietary information of the California sea lion (Zalophus californianus) assessed using stable isotope analysis. Mar Mamm Sci. 2012;28(4):714–32.

There is an error in reference 79. The correct reference is: Polischuk SC, Hobson KA, Ramsay MA. Use of stable-carbon and nitrogen isotopes to assess weaning and fasting in female polar bears and their cubs. Can J Zool. 2001;79(3):499–511.

There is an error in reference 80. The correct reference is: Jenkins SG, Partridge ST, Stephenson TR, Farley SD, Robbins CT. Nitrogen and carbon isotope fractionation between mothers, neonates, and nursing offspring. Oecologia. 2001;129(3):336–41. https://doi.org/10.1007/s004420100755 PMID: 28547188

There is an error in reference 81. The correct reference is: Tsutaya T, Yoneda M. Reconstruction of breastfeeding and weaning practices using stable isotope and trace element analyses: a review. Am J Phys Anthropol. 2015;156(Suppl 59):2–21. https://doi.org/10.1002/ajpa.22657 PMID: 25407359

There is an error in reference 82. The correct reference is: Crowell-Davis SL, Houpt KA, Carnevale J. Feeding and drinking behavior of mares and foals with free access to pasture and water. J Anim Sci. 1985;60(4):883–9. https://doi.org/10.2527/jas1985.604883x PMID: 3988655

There is an error in reference 83. The correct reference is: McKinney T, Smith TW. Diets of adults and lambs of desert bighorn sheep during years of varying rainfall in central Arizona. Southwest Nat. 2007;52(4):520–7

There is an error in reference 84. The correct reference is: Balasse M. Reconstructing dietary and environmental history from enamel isotopic analysis: time resolution of intra‐tooth sequential sampling. Int J Osteoarchaeol. 2002;12(3):155–65

There is an error in reference 85. The correct reference is: Balasse M, Obein G, Ughetto‐Monfrin J, Mainland I. Investigating seasonality and season of birth in past herds: a reference set of sheep enamel stable oxygen isotope ratios. Archaeometry. 2011;54(2):349–68. https://doi.org/10.1111/j.1475-4754.2011.00624.x

There is an error in reference 86. The correct reference is: Spencer F, Verostick K, Serna A, Stantis C, Bowen GJ. Effects of particle size, storage conditions, and chemical pretreatments on carbon and oxygen isotopic measurements of modern tooth enamel. Sci Justice. 2024;64(2):193–201. https://doi.org/10.1016/j.scijus.2024.01.004 PMID: 38431376

There is an error in reference 87. The correct reference is: Varkuleviciute K, Gron KJ, Patterson WP, Panelli C, Rossi S, Timsic S. Transhumance in the Early Neolithic? Carbon and oxygen isotope insights into sheep husbandry at Arene Candide, Northern Italy. J Archaeol Sci Rep. 2021;40:103240

There is an error in reference 88. The correct reference is: Cerling TE, Bernasconi SM, Hofstetter LS, Jaggi M, Wyss F, Rudolf von Rohr C. CH4/CO2 ratios and carbon isotope enrichment between diet and breath in herbivorous mammals. Front Ecol Evolution. 2021;9:638568.

There is an error in reference 89. The correct reference is: Turner E. Large Mammalian Fauna. In: Barton RNE, Bouzouggar A, Collcutt SN, Humphrey LT, editors. Cemeteries and Sedentism in the Later Stone Age of NW Africa: Excavations at Grotte des Pigeons, Taforalt, Morocco. Römisch-Germanischen Zentralmuseums; 2019: 239–308.

There is an error in reference 90. The correct reference is: Segura A, Moreno E. Foraging habitat use by sympatric Cuvier’s Gazelle, Dama Gazelle, and Dorcas Gazelle on a private reserve in Morocco. J Mammal. 2024;105(6):1345–52. https://doi.org/10.1093/jmammal/gyae079

There is an error in reference 91. The correct reference is: Brooks JR, Flanagan LB, Buchmann N, Ehleringer JR. Carbon isotope composition of boreal plants: functional grouping of life forms. Oecologia. 1997;110(3):301–11. https://doi.org/10.1007/s004420050163 PMID: 28307218

There is an error in reference 92. The correct reference is: Kelly CK, Woodward FI. Ecological correlates of carbon isotope composition of leaves: a comparative analysis testing for the effects of temperature, CO2 and O2 partial pressures and taxonomic relatedness on δ13C. J Ecol. 1995:509–15

There is an error in reference 93. The correct reference is: Weiguo L, Xiahong F, Youfeng N, Qingle Z, Yunning C, Zhisheng AN. δ13C variation of C3 and C4 plants across an Asian monsoon rainfall gradient in arid northwestern China. Glob Chang Biol. 2005;11(7):1094–100.

There is an error in reference 94. The correct reference is: Pate FD, Krull E. Carbon isotope discrimination by C3 pasture grasses along a rainfall gradient in South Australia: implications for palaeoecological studies. Quaternary Australasia. 2007;14:29–33

There is an error in reference 95. The correct reference is: Murphy BP, Bowman DM. The carbon and nitrogen isotope composition of Australian grasses in relation to climate. Funct Ecol. 2009;23(6):1040–9.

There is an error in reference 96. The correct reference is: Körner C, Farquhar GD, Roksandic Z. A global survey of carbon isotope discrimination in plants from high altitude. Oecologia. 1988;74(4):623–32. https://doi.org/10.1007/BF00380063 PMID: 28311772

There is an error in reference 97. The correct reference is: Liu X, Zhao L, Gasaw M, Gao D, Qin D, Ren J. Foliar δ13C and δ15N values of C3 plants in the Ethiopia Rift Valley and their environmental controls. Chin Sci Bull. 2007;52:1265–73

There is an error in reference 98. The correct reference is: Wengler L, Vernet J-L. Vegetation, sedimentary deposits and climates during the Late Pleistocene and Holocene in eastern Morocco. Palaeogeogr Palaeoclimatol Palaeoecol. 1992;94(123–4):141–67. https://doi.org/10.1016/0031-0182(92)90117-n

There is an error in reference 99. The correct reference is: Nardini A, Salleo S, Lo Gullo MA, Pitt F. Different responses to drought and freeze stress of Quercus ilex L. growing along a latitudinal gradient. Plant Ecol. 2000;148(2):139–47. https://doi.org/10.1023/a:1009840203569

There is an error in reference 100. The correct reference is: Reade H, O’Connell TC, Barker G, Stevens RE. Pleistocene and Holocene palaeoclimates in the Gebel Akhdar (Libya) estimated using herbivore tooth enamel oxygen isotope compositions. Quat Int. 2016;404:150–62.

There is an error in reference 101. The correct reference is: Binford LR. Willow smoke and dogs’ tails: hunter-gatherer settlement systems and archaeological site formation. Am Antiq. 1980;45(1):4–20.

There is an error in reference 102. The correct reference is: Rowley-Conwy P, Zvelebil M. Saving it for later: storage by prehistoric hunter-gatherers in Europe. In: Halstead P, O’Shea J, editors. Bad year economics: cultural responses to risk and uncertainty. Cambridge University Press; 1989: 40–56.

There is an error in reference 103. The correct reference is: Tushingham S, Bettinger RL. Why foragers choose acorns before salmon: Storage, mobility, and risk in aboriginal California. J Anthropol Archaeol. 2013;32(4):527–37.

There is an error in reference 104. The correct reference is: Hofman JL. Hunter-gatherer mortuary variability: toward an explanatory model. University of Tennessee; 1986

There is an error in reference 105. The correct reference is: Littleton J, Allen H. Hunter-gatherer burials and the creation of persistent places in southeastern Australia. J Anthropol Archaeol. 2007;26(2):283–98.

There is an error in reference 106. The correct reference is: Munro N. Zooarchaeological measures of hunting pressure and occupation intensity in the Natufian: implications for agricultural origins. Curr Anthropol. 2004;45(S4):S5–34.

There is an error in reference 107. The correct reference is: Worthey KB, Stiner MC, Quade J, Rowland JC, Açıkkol A, Baykara I, et al. Paleolithic Human Responses to Changing Aridity at Üçağızlı I cave, southern-coastal Turkey: Application of a Novel Carbon Isotope-Based Method. J Archaeol Method Theory. 2022;29(4):1190–228. https://doi/org/10.1007/s10816-022–09553-x

There is an error in reference 108. The correct reference is: Kelly RL. The lifeways of hunter-gatherers: the foraging spectrum. Cambridge University Press; 2013.

There is an error in reference 109. The correct reference is: Stiner M, Munro N, Surovell T, Tchernov E, Bar-Yosef O. Paleolithic population growth pulses evidenced by small animal exploitation. Science. 1999;283(5399):190–4. https://doi.org/10.1126/science.283.5399.190 PMID: 9880245

There is an error in reference 110. The correct reference is: Richerson PJ, Boyd R, Bettinger RL. Was agriculture impossible during the Pleistocene but mandatory during the Holocene? A climate change hypothesis. Am Antiq. 2001;66(3):387–411.

There is an error in reference 111. The correct reference is: Jeffrey A. Exploring palaeoaridity using stable oxygen and carbon isotopes in small mammal teeth: a case study from two Late Pleistocene archaeological cave sites in Morocco, North Africa. Doctoral dissertation. University of Oxford; 2016.

**Table 1:** Sampled contexts from Taforalt including the full range of ^14^C and OSL ages associated with each layer and assigned temporal intervals.

Sector	Layers	Date range (cal. years BP)	Dating method	Reference	Assigned temporal interval (this paper)	Notes
8	L3 (Grey series)	12,700-12,817	^14^C (n=1). 2σ range of modeled posterior age	[58]	early Younger Dryas	
8	L6-L29/G100 (Grey series)	13,169-14,970	^14^C (n=24). 2σ range of modeled posterior ages	[58]	GI-1 (Bølling-Allerød)	Sector 10 data exclude one sample (OxA-29264) with a modeled 2σ error range that does not overlap with the other samples
10	Grey series	13,993-15,211	^14^C (n=16). 2σ range of ages	[58]		
10	Brown	Intermediate in age between Sector 10 grey series and Sector 13 orange layer	–	L. Humphrey personal communication (2024)	–	Although grouped here, differences in dental micro- and mesowear between these two layers may indicate they were deposited at slightly different times (Uzunidis et al., 2022)
13	Dark Brown					
8	Y1	14,855-15,615	^14^C (n=4). 2σ range of modeled posterior ages	[58]	Heinrich Stadial 1	Excludes one sample (TAF08-6836) with a modeled 2σ error range that does not overlap with the other samples from layer Y1
13	Orange	17,569-17,982	^14^C (n=1). 1σ range	[55]	Heinrich Stadial 1	^14^C date insecure given low collagen yield.
8	Y2	16,745-20,136	^14^C (n=10). 2σ range of modeled posterior ages	[58]	Heinrich Stadial 1 to late LGM	
8	Y4	19,940-24,635	^14^C (n=3). 2σ range of modeled posterior ages	[58]	LGM	
2	R7-R10	20,370-52,300	^14^C (n=4) and OSL (n=1). 1σ range of ages. Layers bracketed and not directly dated.	[59]	LGM or MIS 3	
8	Y8-Y13	30,880-34522	^14^C (n=3). 2σ range of modeled posterior ages. Layers bracketed and not directly dated.	[9]	MIS 3	
2	R16-R17	56,200-64,000	OSL (n=1). 1σ range	[59]	MIS 4	
2	R19-R20	56,200-88,900	OSL (n=2). 1σ range. Layers bracketed and not directly dated.	[59]	MIS 4 or MIS 5a-b	
2	R21-R23	73,400-91,600	OSL (n=1). 2σ range of modeled posterior age	[46]	MIS 5a-b	

**Fig 1:**
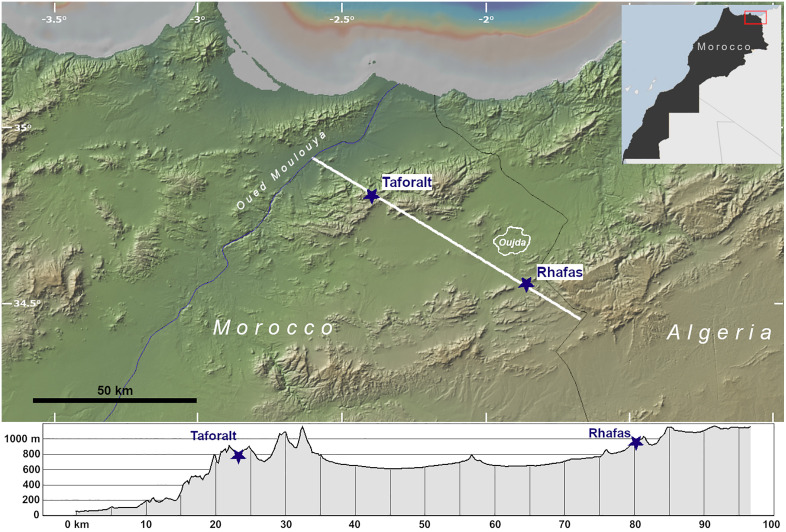
Locations of Taforalt and Rhafas caves in northeastern Morocco. The elevation profile is derived from a NW-SE transect indicated on the map by a white line, which runs from Oued Moulouya to the Algerian border and cross-cuts Taforalt and Rhafas Caves (blue stars). Figure made with GeoMapApp (www.geomapapp.org)/ CC BY/ CC BY [52].

**Fig 2:**
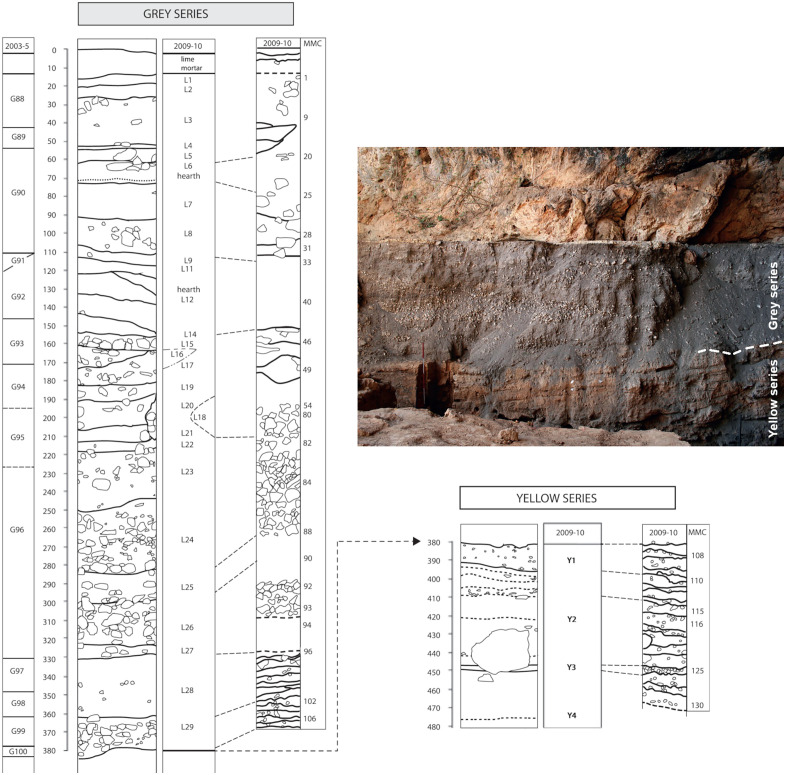
Taforalt Sector 8 stratigraphy of the Grey Series and Yellow Series. Figure modified from Humphrey et al. [18] S1 Fig and Collcutt [56] Fig 2.15. Photograph by Ian R. Cartwright.
