# Supplementary figures and images for: Expansion of forest cover and coeval shifts in Later Stone Age land-use at Taforalt and Rhafas Caves, Morocco, as inferred from carbon isotopes in ungulate tooth enamel
Source: PLoS One. 2025 Jun 12;20(6):e0325691. doi: 10.1371/journal.pone.0325691 (PMC12161528; doi:10.1371/journal.pone.0325691)

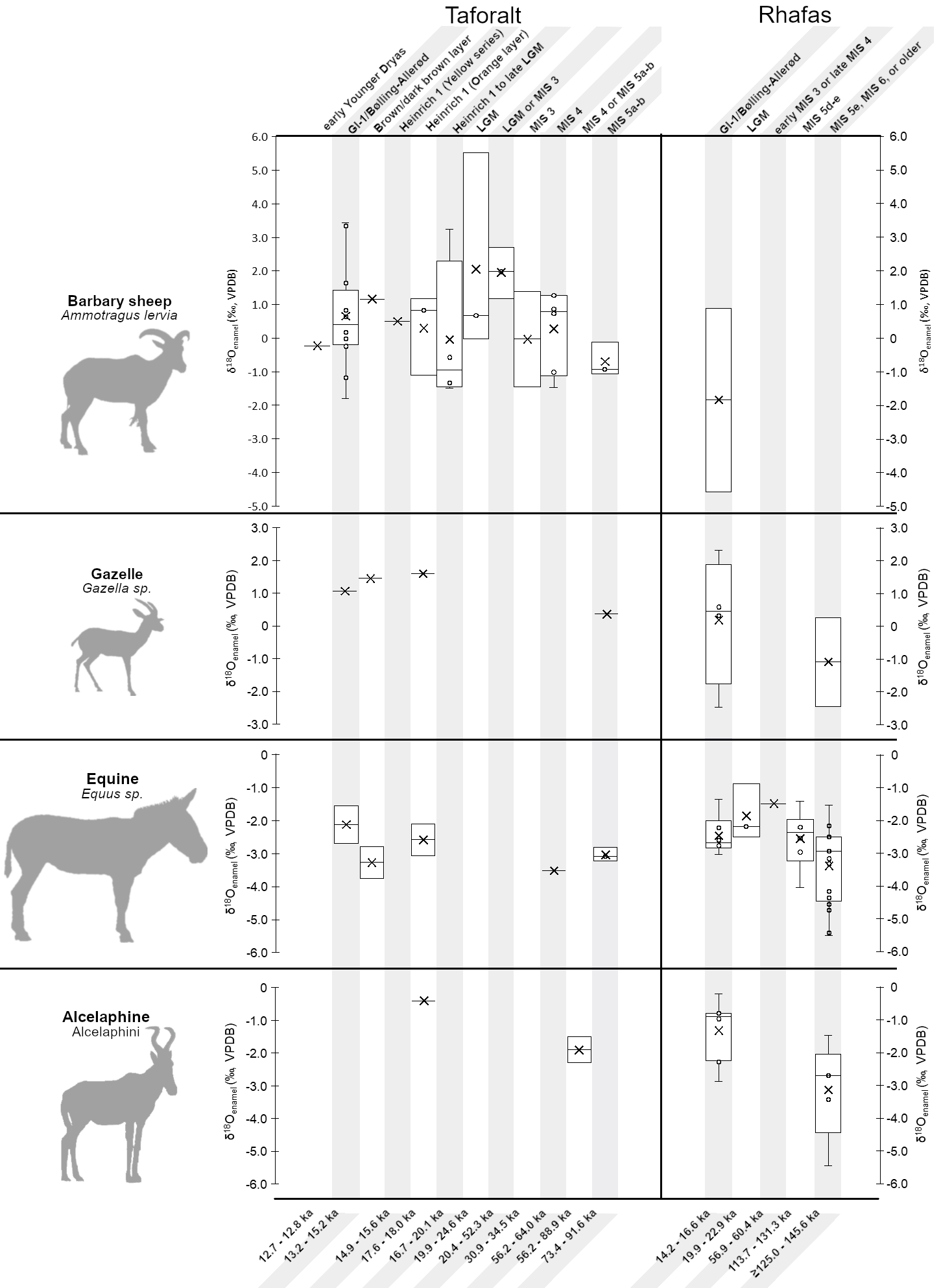

Supplement: S1 Fig — (TIF) [file pone.0325691.s004.tif]
