# Supplementary material for: Expansion of forest cover and coeval shifts in Later Stone Age land-use at Taforalt and Rhafas Caves, Morocco, as inferred from carbon isotopes in ungulate tooth enamel
Source: PLoS One. 2025 Jun 12;20(6):e0325691. doi: 10.1371/journal.pone.0325691 (PMC12161528; doi:10.1371/journal.pone.0325691)

S1 File. Plots of intra-tooth $\delta^{13}C_{enamel}$ (black) and $\delta^{18}O_{enamel}$ (blue) values for each sequentially sampled specimen.

Plots are organized by taxon, and sequentially from youngest to oldest stratigraphic context.

Barbary sheep (*Ammotragus lervia*)

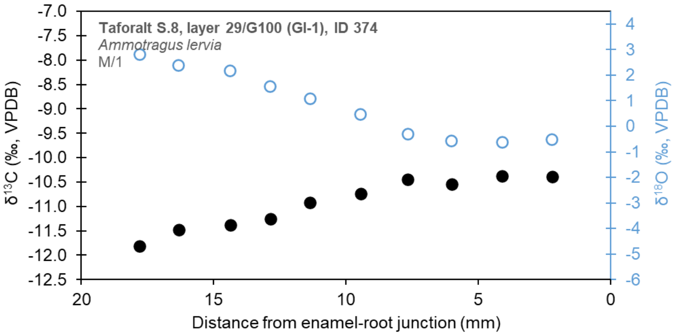

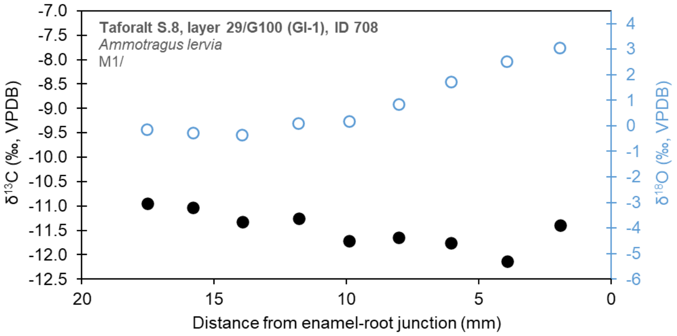

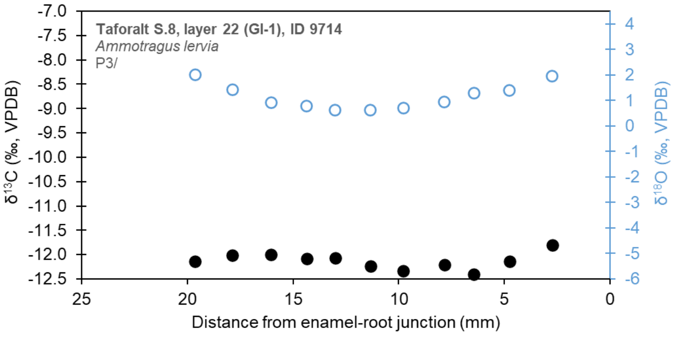

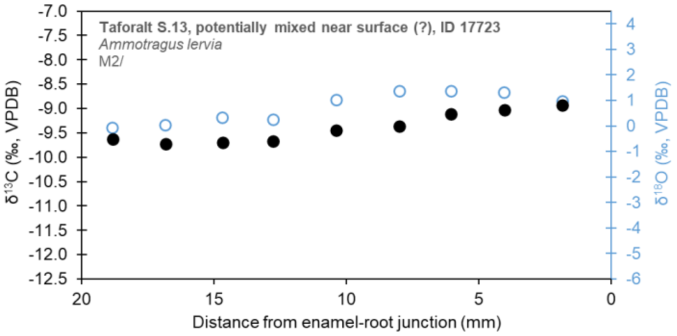

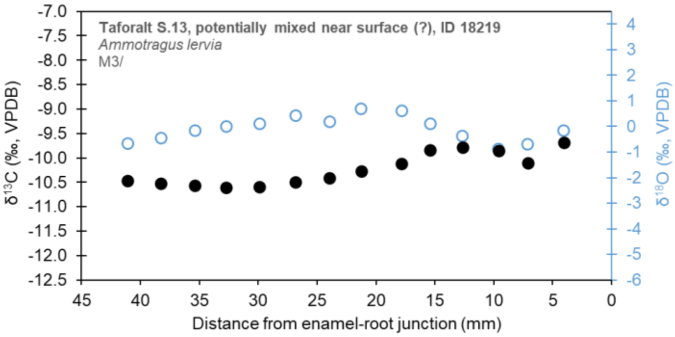

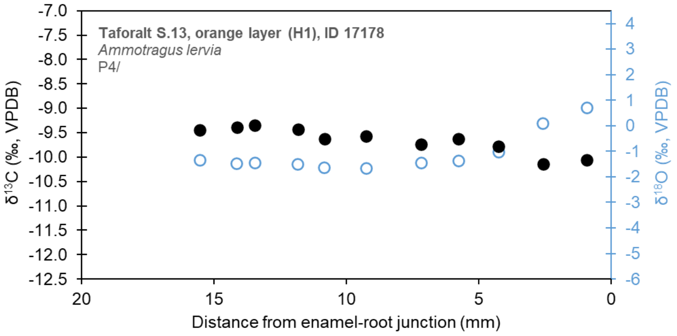

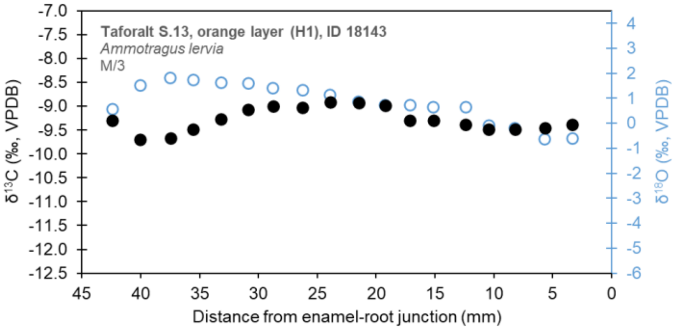
Taforalt S.13, orange layer (H1), ID 18143
Ammotragus lervia
M/3
δ13C (‰, VPDB)
δ18O (‰, VPDB)
Distance from enamel-root junction (mm)

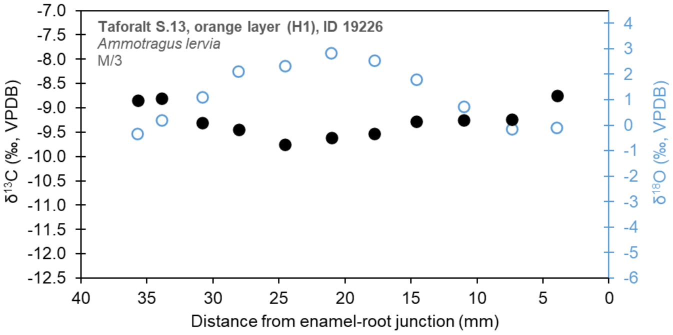
Taforalt S.13, orange layer (H1), ID 19226
Ammotragus lervia
M/3
δ13C (‰, VPDB)
δ18O (‰, VPDB)
Distance from enamel-root junction (mm)

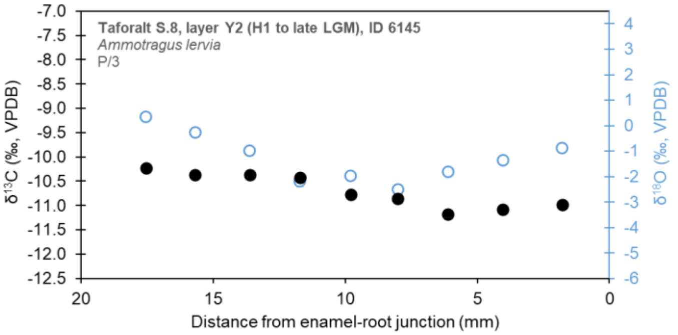
Taforalt S.8, layer Y2 (H1 to late LGM), ID 6145
Ammotragus lervia
P/3
δ13C (‰, VPDB)
δ18O (‰, VPDB)
Distance from enamel-root junction (mm)

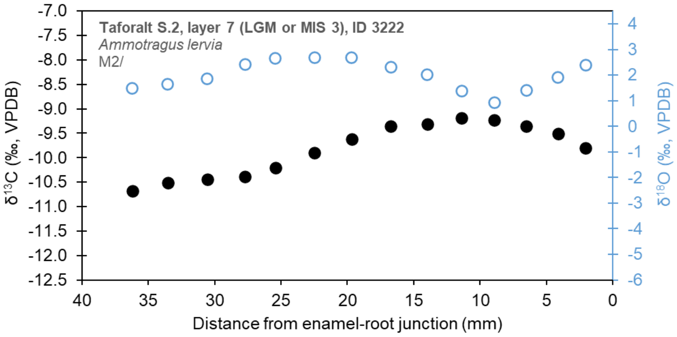
Taforalt S.2, layer 7 (LGM or MIS 3), ID 3222
Ammotragus lervia
M2/
δ13C (‰, VPDB)
δ18O (‰, VPDB)
Distance from enamel-root junction (mm)

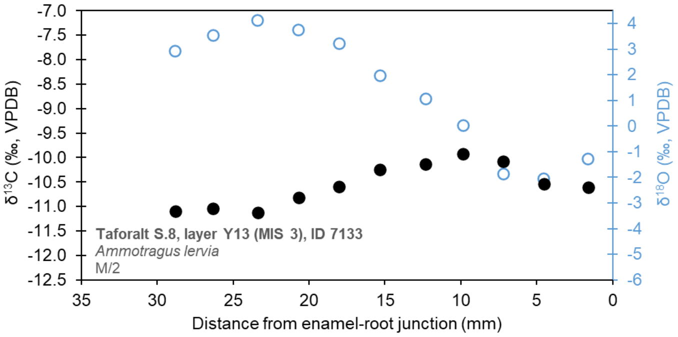
Taforalt S.8, layer Y13 (MIS 3), ID 7133
Ammotragus lervia
M/2
δ13C (‰, VPDB)
δ18O (‰, VPDB)
Distance from enamel-root junction (mm)

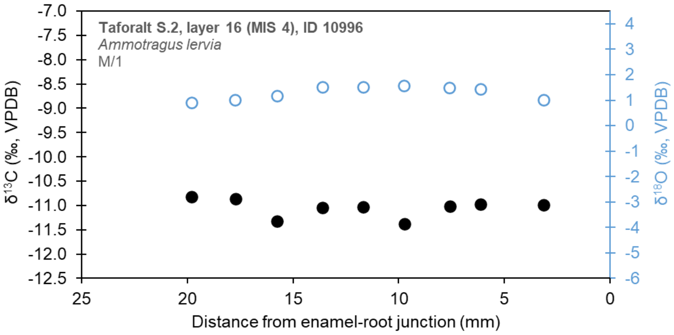
Taforalt S.2, layer 16 (MIS 4), ID 10996
Ammotragus lervia
M/1
δ13C (‰, VPDB)
δ18O (‰, VPDB)
Distance from enamel-root junction (mm)

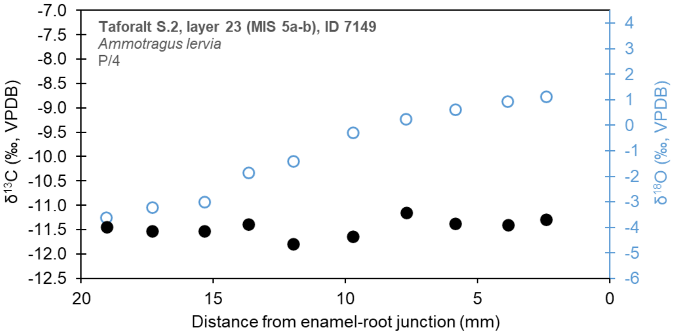
Taforalt S.2, layer 23 (MIS 5a-b), ID 7149
Ammotragus lervia
P/4
δ13C (‰, VPDB)
δ18O (‰, VPDB)
Distance from enamel-root junction (mm)

Equines (*Equus* sp., *Equus africanus/asinus, Equus mauritanicus*)

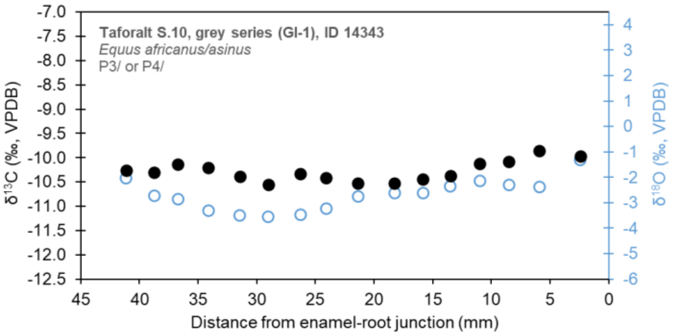

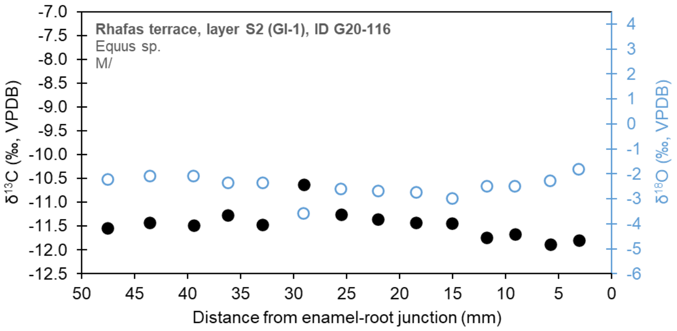

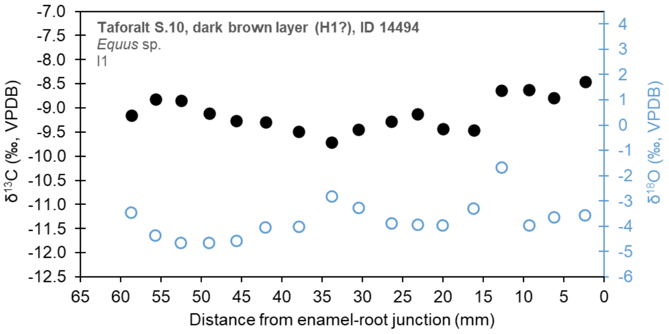

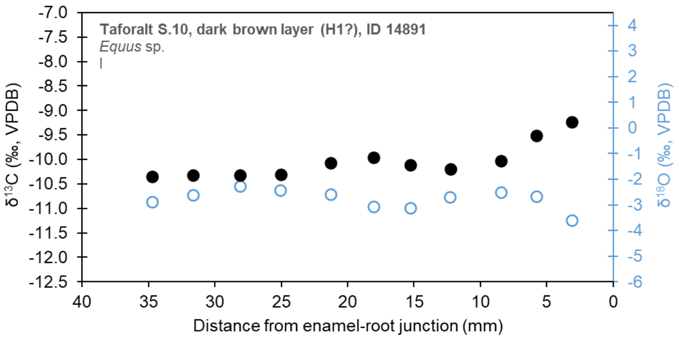

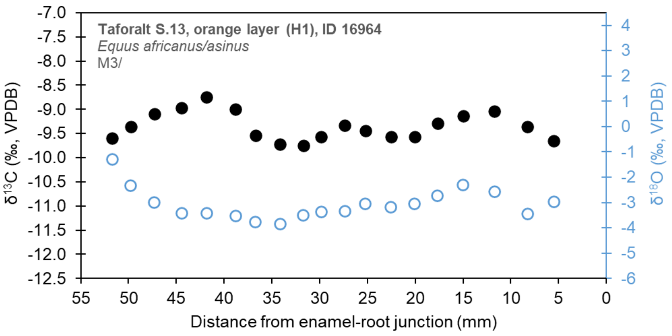

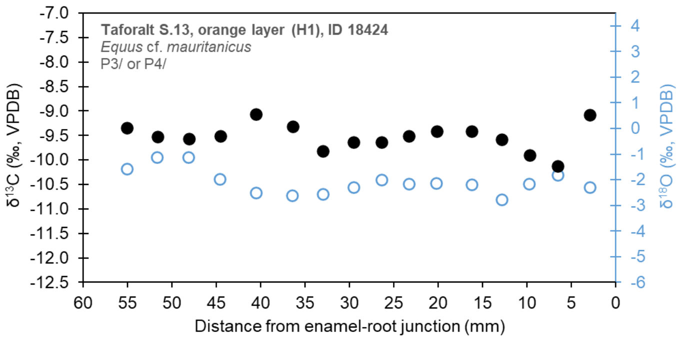

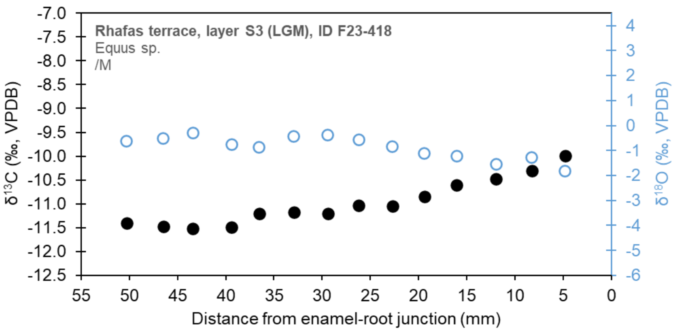

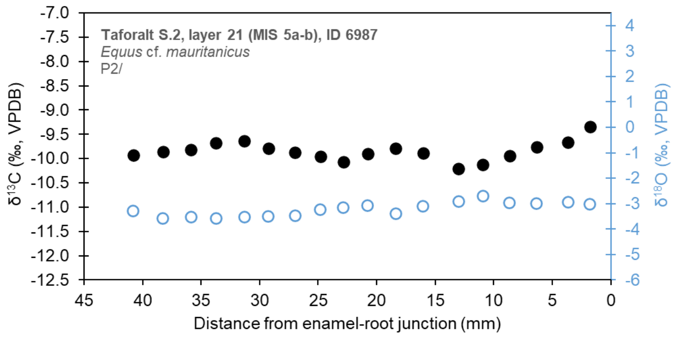

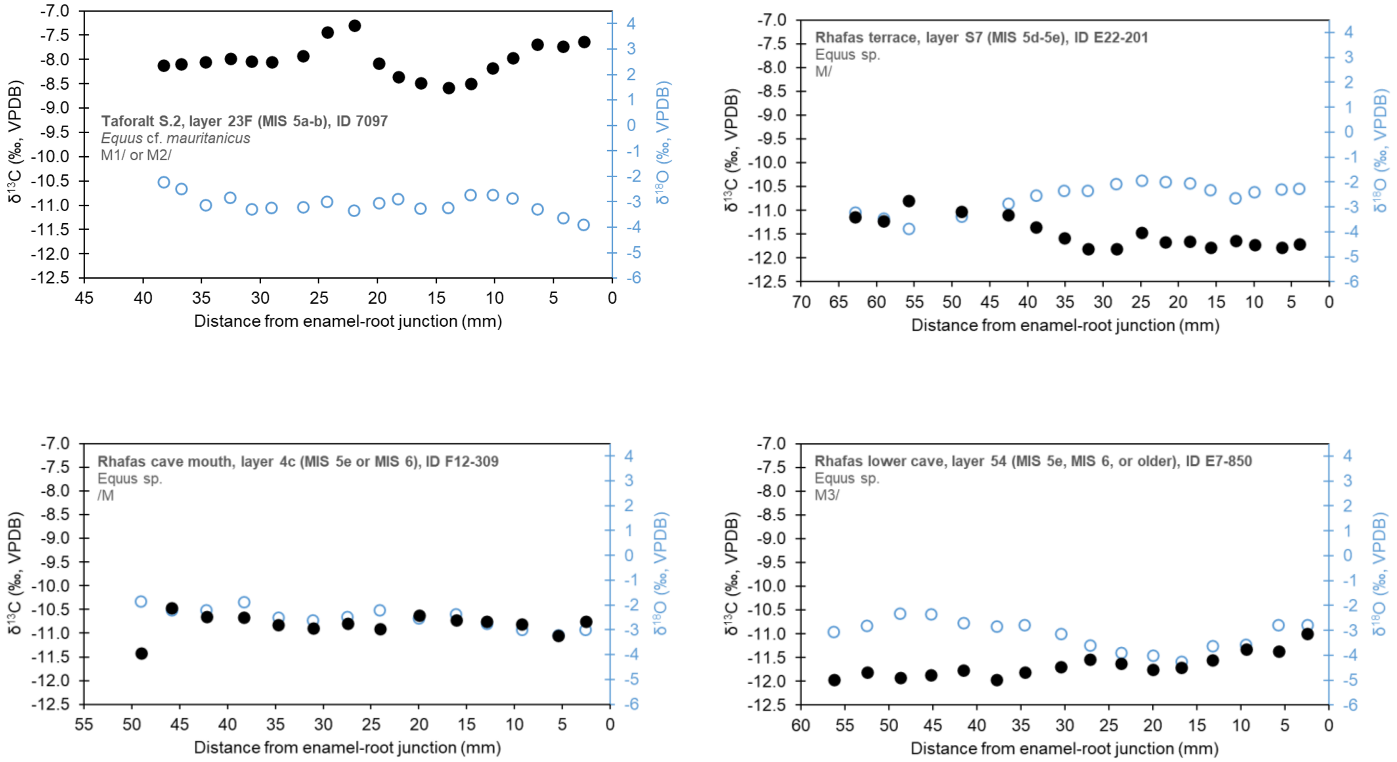
Taforalt S.2, layer 23F (MIS 5a-b), ID 7097
Equus cf. mauritanicus
M1/ or M2/
Rhafas terrace, layer S7 (MIS 5d-5e), ID E22-201
Equus sp.
M/
Rhafas cave mouth, layer 4c (MIS 5e or MIS 6), ID F12-309
Equus sp.
/M
Rhafas lower cave, layer 54 (MIS 5e, MIS 6, or older), ID E7-850
Equus sp.
M3/
δ13C (‰, VPDB)
δ18O (‰, VPDB)
Distance from enamel-root junction (mm)

Alcelaphines (Alcelaphini, *Alcelaphus buselaphus*)

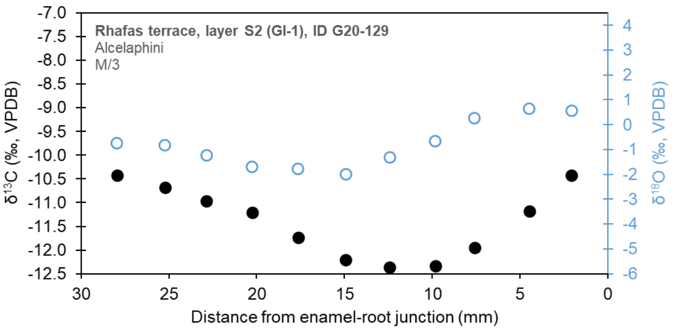

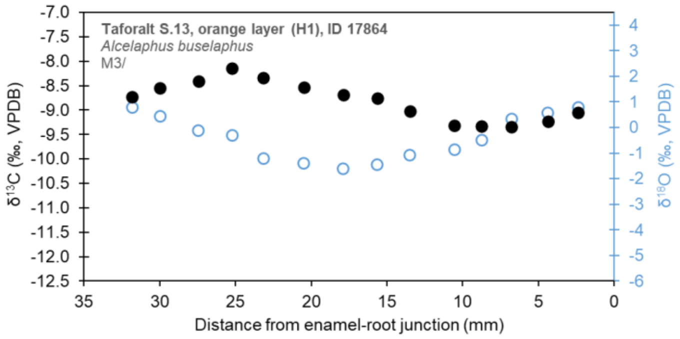

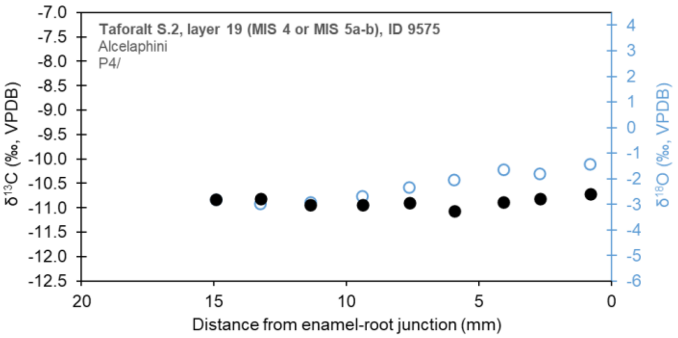

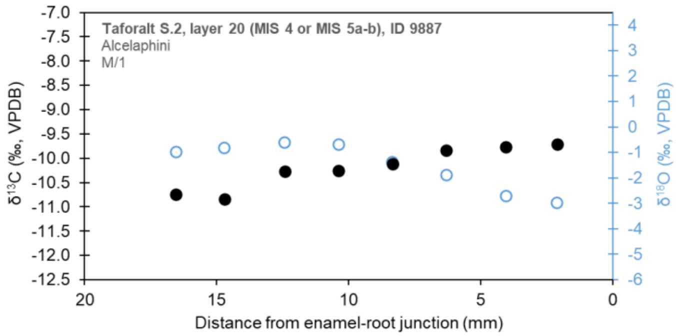

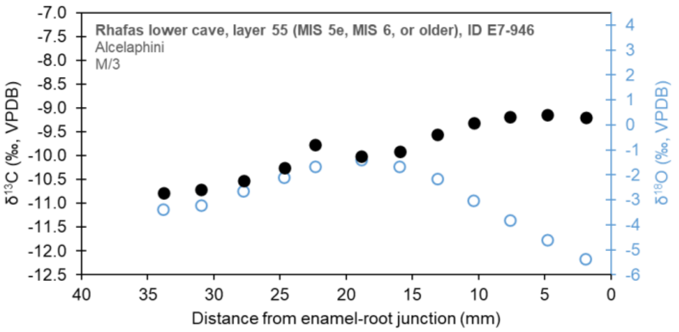

Supplement: S1 File — (PDF) [file pone.0325691.s005.pdf]
